# The type I FLT3 inhibitor XL999 overrides secondary FLT3-ITD mutations that arise under gilteritinib and quizartinib treatment in acute myeloid leukemia

**DOI:** 10.1186/s43556-026-00561-x

**Published:** 2026-09-04

**Authors:** Shiyang Wang, Yvyin Zhang, Zhiwei Zhong, Jiajun He, Jiaqi Fan, Qi Xiang, Fangfang Yang, Yuping Zhang, Shunqing Wang, Peihong Wang

**Affiliations:** 1 Department of Hematology, Guangzhou First People’s Hospital, School of Medicine, South China University of Technology, Guangzhou, 510000 P.R. China; 2 Department of Hematology, Guangdong Provincial People’s Hospital, School of Medicine, South China University of Technology, Guangzhou, 510000 P.R. China; 3 Department of Hematologic Oncology, Sun Yat-Sen University Cancer Center, Guangzhou, P.R. China

**Keywords:** XL999, FLT3-ITD, Acute myeloid leukemia, FLT3 resistance mutation, Gilteritinib, Quizartinib

## Abstract

FMS-like tyrosine kinase 3 (FLT3) internal tandem duplication (FLT3-ITD) is a well-characterized genetic alteration associated with poor prognosis in acute myeloid leukemia (AML). Although FLT3 inhibitors, such as gilteritinib and quizartinib, initially show clinical efficacy, resistance frequently emerges because of secondary mutations. Through a kinase inhibitor library screen, we identified XL999, which exhibits potent antileukemic activity across diverse FLT3-mutant AML cell lines and primary patient samples. Mechanistically, biochemical and molecular docking analyses revealed that XL999 directly binds to FLT3-ITD, potentially independent of the common F691 resistance site, thereby suppressing downstream signaling pathways to induce cell cycle arrest and apoptosis. In preclinical mouse models harboring refractory FLT3-ITD-D835Y or ITD-F691L mutations, oral administration of XL999 effectively reduced the leukemic burden across the peripheral blood, spleen, and bone marrow, significantly prolonging survival compared with gilteritinib and quizartinib, with no overt systemic toxicity observed in short-term mouse tolerability studies. Furthermore, XL999 demonstrated robust efficacy in patient-derived xenograft models, complemented by a favorable oral bioavailability profile. Together, these findings suggest that XL999 may represent an orally bioavailable FLT3 inhibitor with the potential to overcome clinically relevant secondary resistance mutations in FLT3-ITD-positive AML, supporting its further investigation as a therapeutic candidate.

**Supplementary Information:**

The online version contains supplementary material available at https://doi.org/10.1186/s43556-026-00561-x.

## Introduction

Receptor tyrosine kinases (RTKs) are critical regulators of cell growth, differentiation, and developmental signaling, and their dysregulation is frequently implicated in human cancers [1]. Among them, activating mutations in FMS-like tyrosine kinase 3 (FLT3) represent one of the most common genetic alterations in acute myeloid leukemia (AML) [2, 3]. FLT3 mutations primarily occur as internal tandem duplications (ITDs) or tyrosine kinase domain (TKD) mutations. FLT3-ITD, caused by insertions of 3–1,236 base pairs within the juxtamembrane domain, is detected in approximately 25% of patients with AML and is strongly associated with poor prognosis [4–6], whereas TKD mutations occur in approximately 7% of patients, and their prognostic significance remains unclear [7, 8]. Mechanistically, FLT3-ITD mutations disrupt the autoinhibitory function of the juxtamembrane domain, promote a conformational transition of the activation loop from the DFG (Asp-Phe-Gly motif)-out state to the DFG-in state, and thereby facilitate constitutive FLT3 activation [9–11]. The persistent activation of downstream signaling pathways, including Janus kinase/signal transducer and activator of transcription 5 (JAK/STAT5), mitogen-activated protein kinase (MAPK), and phosphatidylinositol 3-kinase/protein kinase B (PI3K/AKT), ultimately drives leukemogenesis by promoting proliferation, blocking differentiation, and suppressing the apoptosis of leukemic cells [12–14].

Given the poor prognosis associated with FLT3-ITD-positive AML, considerable efforts have been made to develop FLT3-targeted therapies [15]. First-generation FLT3 inhibitors, including midostaurin, sorafenib, sunitinib, lestaurtinib, and tandutinib, are multitarget kinase inhibitors with limited selectivity and are frequently associated with suboptimal efficacy, off-target toxicity, and treatment resistance [16–20]. Second-generation inhibitors, such as gilteritinib, quizartinib, and crenolanib, were subsequently developed to achieve more potent and selective FLT3 inhibition [8, 21, 22]. Midostaurin in combination with intensive chemotherapy was approved by the U.S. Food and Drug Administration (FDA) in 2017 for newly diagnosed FLT3-mutated AML, followed by the approval of quizartinib in 2023 for newly diagnosed FLT3-ITD-positive AML [23, 24]. Nevertheless, gilteritinib remains the only globally approved monotherapy for patients with relapsed or refractory FLT3-mutated AML [25]. Despite these therapeutic advances, acquired drug resistance and disease relapse remain major barriers to durable clinical responses [8, 26].

Multiple mechanisms contribute to resistance to FLT3 inhibitors, including increased FLT3 ligand production within the bone marrow (BM) microenvironment, CYP3A4-mediated drug metabolism by BM stromal cells, persistent activation of downstream signaling pathways, and acquired mutations in alternative oncogenic pathways [27–31]. Among these mechanisms, secondary mutations within the FLT3 tyrosine kinase domain, particularly substitutions at D835, Y842, and the gatekeeper residue F691, represent the most clinically relevant causes of resistance. These mutations are especially common following treatment with type II FLT3 inhibitors, which preferentially bind the inactive DFG-out conformation of FLT3. For example, all eight patients who relapsed after quizartinib therapy in one study acquired polyclonal or monoclonal FLT3-TKD mutations involving the F691, D835, or Y842 residues [32]. Although the type I inhibitor gilteritinib retains activity against several secondary mutations, including D835 and Y842, no approved therapy effectively targets the clinically important F691L gatekeeper mutation [33, 34]. Therefore, identifying novel FLT3 inhibitors capable of overcoming secondary resistance mutations remains an important unmet clinical need.

To address this challenge, we screened a subset of the MedChemExpress (MCE) kinase inhibitor library (400 of 2,807 compounds) and identified XL999 as a potential FLT3 inhibitor. XL999 is a spectrum-selective kinase inhibitor that targets fibroblast growth factor receptor 1 (FGFR1), vascular endothelial growth factor receptor 2 (VEGFR2), platelet-derived growth factor receptor (PDGFR), FLT3, rearranged during transfection (RET), and SRC proto-oncogene, non-receptor tyrosine kinase (SRC) [35]. Previous clinical studies have demonstrated the preliminary antitumor activity of XL999, particularly in patients with FLT3-ITD-positive AML [36–38]. However, the molecular mechanism underlying FLT3 inhibition, activity against clinically relevant secondary FLT3 resistance mutations, and the pharmacokinetic characteristics and therapeutic potential of orally administered XL999 remain largely unknown.

We hypothesized that XL999 functions as a type I FLT3 inhibitor capable of overcoming clinically relevant secondary FLT3 resistance mutations, particularly F691L. To test this hypothesis, we comprehensively evaluated the kinase selectivity, molecular binding mechanism, antileukemic activity, efficacy against FLT3 secondary mutations, oral pharmacokinetic properties, and preliminary safety profile of XL999 using biochemical assays, cellular models, primary AML samples, patient-derived xenograft models, and pharmacokinetic analyses. Collectively, our findings indicated that XL999 effectively inhibited FLT3 signaling, retained potent activity against clinically relevant FLT3 resistance mutations, and exhibited favorable oral pharmacokinetic characteristics, supporting its further investigation as a potential orally administered therapeutic candidate for FLT3-ITD-positive AML.

## Results

### Identification of XL999 as a potent FLT3 inhibitor

**Fig. 1:**
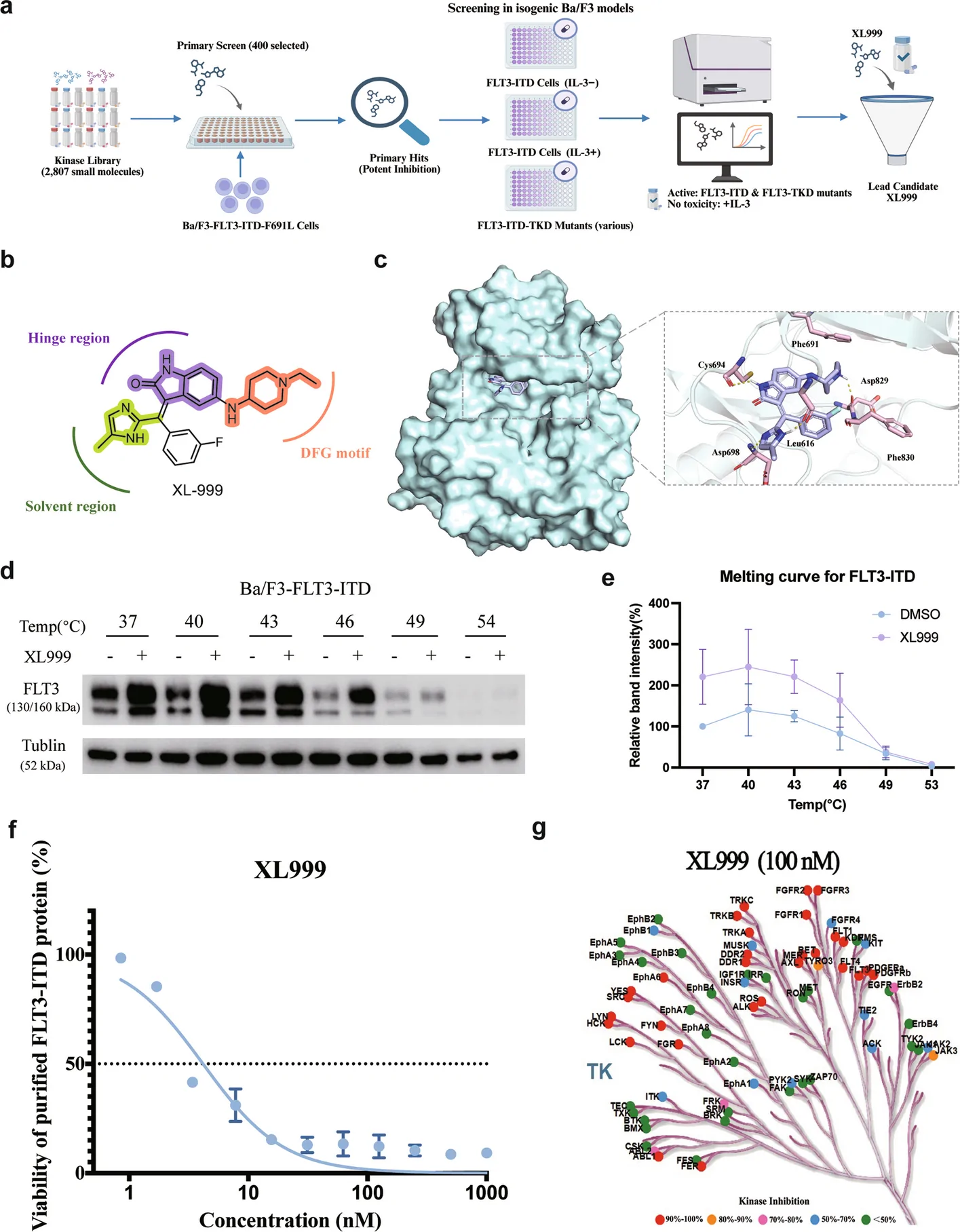
Identification and characterization of XL999 as a potent FLT3 inhibitor. **a** High-throughput screening workflow of a 400-compound subset derived from a 2,807-member kinase inhibitor library against Ba/F3-FLT3-ITD-F691L cells, followed by screening in isogenic Ba/F3 models for lead compound identification. Schematic diagrams were created with BioRender.com (Agreement number: FMZXJD06KP). **b** Chemical structure of XL999. **c** Molecular docking model of XL999 bound to FLT3 (PDB code: 6JQR). Predicted hydrogen-bond interactions with Cys694, Leu616, Asp698, and Asp829 are shown, highlighting the absence of interaction with the gatekeeper residue Phe691. **d** Representative Western blot from a cellular thermal shift assay (CETSA) in Ba/F3-FLT3-ITD cells treated with DMSO or XL999 (1 μM) across a temperature gradient (37–54 °C). Tubulin was used as the loading control. **e** Quantification of CETSA-derived thermal stability curves based on immunoblot band intensities. Data are presented as mean ± SD from three independent experiments. **f** In vitro dose–response inhibition curve of XL999 against purified FLT3-ITD protein assessed using an ADP-Glo (IC_50_ = 4.159 nM). **g** Kinase selectivity profile of XL999 at 100 nM. Kinome tree representation of inhibitory activity across the human tyrosine kinase (TK) family. Colored circles indicate discrete ranges of percentage kinase inhibition

To identify therapeutic agents capable of effectively targeting FLT3 and overcoming secondary resistance mutations, we screened a subset of 400 compounds from a 2,807-member small-molecule library using Ba/F3 cells expressing FLT3-ITD-F691L. At an initial screening concentration of 100 nM, several molecules, including ATH686, 2,4,6-trihydroxybenzoic acid, XL999, and sitravatinib, demonstrated marked antileukemic activity (Fig. S1). Given that ATH686 and sitravatinib have been reported to be effective FLT3 inhibitors [39, 40], 2,4,6-trihydroxybenzoic acid and XL999 were selected for further evaluation. They were evaluated at concentrations ranging from 0.15 to 1,000 nM against a panel of isogenic Ba/F3 models, including FLT3-ITD cells [with or without 1 nM interleukin-3 (IL-3)] and FLT3-ITD-D835Y/F691L mutants. Candidates were prioritized if they exhibited robust inhibitory activity against both FLT3-ITD and the tested resistant mutants but showed no cytotoxicity toward IL-3-supplemented cells (thereby confirming selectivity for FLT3-dependent growth). The 2,4,6-trihydroxybenzoic acid failed to exhibit an antileukemic effect during this validation stage, whereas XL999 was successfully identified as the optimal lead candidate (Fig. 1a, Fig. S2a).

To investigate the potential binding mode of XL999 within the ATP-binding pocket of FLT3, in silico molecular docking simulations were performed. As modeled in Fig. 1b, c, XL999 was predicted to accommodate the “DFG-in” conformation of FLT3, a hallmark characteristic of typical type I inhibitors. Specifically, the computational model suggested that the indolone moiety of XL999 forms putative hydrogen bonds with the hinge residue Cys694. The planar conformation of the carbon‒carbon double bond extending from the indolone core appears to restrict the orientation of the 5-methyl-1H-indazolyl group, directing it toward the solvent-exposed region. This spatial arrangement theoretically optimizes the torsion angle, allowing the ring to form two distinct hydrogen bonds with Leu616 and Asp698, thereby potentially stabilizing the ligand–receptor complex. Furthermore, the N-ethylpiperidine moiety was modeled to extend toward the DFG motif, establishing a potential hydrogen bond with Asp829. In contrast to gilteritinib (Fig. S2b), which typically maintains its affinity via key bidentate hydrogen bonds formed by its pyrazine carboxamide core, XL999 is proposed to sustain its binding through this distributed hydrogen-bonding network. Notably, the gatekeeper residue Phe691 did not appear to play a critical role in stabilizing the predicted binding pose. Consequently, the simulated binding mode of XL999 with the FLT3-F691L mutant remained virtually identical to that of the wild-type kinase (Fig. S2c), providing a plausible molecular rationale for the robust inhibitory efficacy of XL999 against Ba/F3-FLT3-ITD-F691L cells.

To determine whether XL999 could engage with the FLT3-ITD protein, we performed a cellular thermal shift assay (CETSA) in Ba/F3-FLT3-ITD cells. The results revealed a distinct rightward shift in the melting curve of FLT3-ITD upon treatment with 1000 nM XL999 compared with that of the dimethyl sulfoxide (DMSO) control (Fig. 1d, e). This significant increase in thermal stability confirmed the successful target engagement between XL999 and FLT3-ITD.

To verify whether the cellular effects of XL999 stemmed from its direct interaction with FLT3-ITD, an in vitro ADP-Glo™ kinase assay (ADP-Glo) was performed using purified recombinant proteins. XL999 demonstrated potent, dose-dependent inhibition of FLT3-ITD, yielding an IC_50_ value of 4.16 nM (Fig. 1f).

To systematically characterize the kinase selectivity spectrum of XL999, in vitro screening against a 76-target tyrosine kinase (TK) panel was performed at a concentration of 100 nM (Fig. 1g and Fig. S3). In addition to its primary targets, XL999 exhibited robust inhibitory activity against AXL receptor tyrosine kinase (AXL), RET, and SRC family kinases, including LYN proto-oncogene, Src family tyrosine kinase (LYN) and lymphocyte-specific protein tyrosine kinase (LCK). Furthermore, marked inhibition of VEGFR2 and FGFR1 kinase activity was observed.

### XL999 exhibits potent antileukemic activity in FLT3-ITD-positive cell lines in vitro

**Fig. 2:**
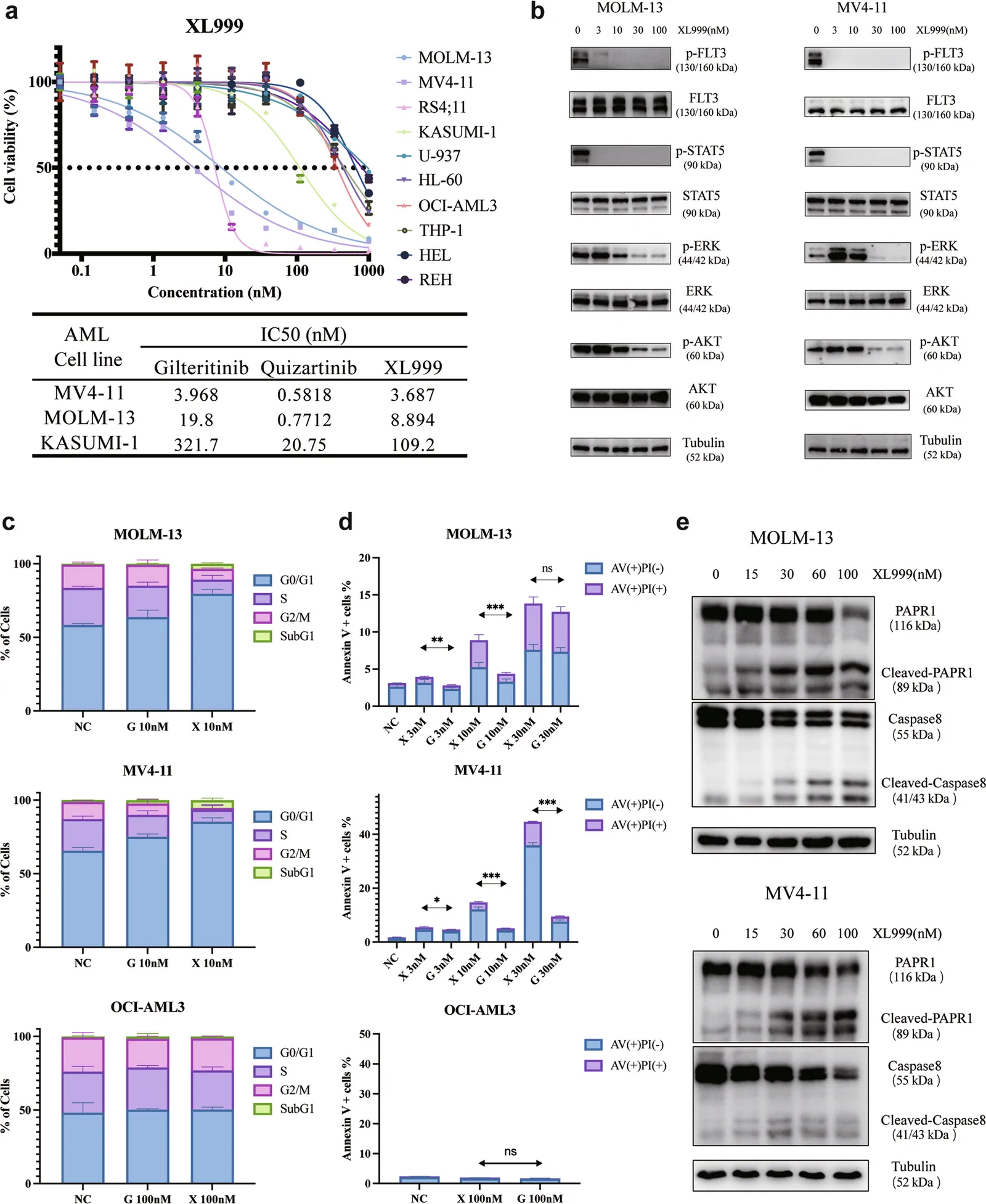
XL999 potently inhibits cell growth and induces apoptosis in FLT3-ITD-positive cells. **a** Dose–response curves of AML cell lines with distinct FLT3 genotypes following 48 h of treatment with XL999. Cell viability was normalized to DMSO-treated controls. The table below summarizes the IC₅₀ values of gilteritinib, quizartinib, and XL999 in FLT3-ITD-positive AML cell lines and the FLT3-ITD-negative AML cell line KASUMI-1. Data are presented as mean ± SD from three independent experiments. **b** Immunoblot analysis of total and phosphorylated (p-) FLT3, STAT5, ERK, and AKT in MOLM-13 and MV4-11 cells following treatment with the indicated concentrations of XL999 (0–100 nM) for 6 h. Tubulin was used as the loading control. **c** Flow cytometric analysis of cell cycle distribution in MOLM-13, MV4-11, and OCI-AML3 cells after 24 h of treatment with vehicle control (NC; DMSO), gilteritinib (G), or XL999 (X) at the indicated concentrations. The percentages of cells in the G₀/G₁, S, G₂/M, and sub-G₁ phases are shown. **d** Quantification of apoptotic cell populations in MOLM-13, MV4-11, and OCI-AML3 cells following 48 h of treatment under the indicated conditions. Apoptosis was assessed by Annexin V/propidium iodide (PI) staining and flow cytometry. Bar graphs show the percentages of early apoptotic (Annexin V⁺/PI⁻) and late apoptotic (Annexin V⁺/PI⁺) cells. **e** Immunoblot analysis of PARP1, caspase-8, and their corresponding cleaved forms in MOLM-13 and MV4-11 cells following 48 h of exposure to increasing concentrations of XL999 (0–100 nM). Tubulin was used as the loading control. Statistical significance is indicated as follows: **P < 0.01, ***P < 0.001; ns, not significant. Abbreviations: X, XL999; G, gilteritinib; NC, vehicle control

The antileukemic efficacy of XL999 was evaluated across a diverse panel of leukemia cells, encompassing FLT3-ITD-positive (MOLM-13 and MV4-11), FLT3-overexpressing (RS4;11), and FLT3-ITD-negative lines (KASUMI-1, U-937, HL-60, OCI-AML3, THP-1, HEL, and REH). XL999 robustly suppressed the viability of the FLT3-driven cell lines but had negligible effects on the viability of the FLT3-ITD-negative AML cells (Fig. 2a). Specifically, XL999 exhibited IC_50_ values of 8.894 nM and 3.687 nM in MOLM-13 and MV4-11 cells, respectively, outperforming gilteritinib (Fig. 2a). Western blot analysis confirmed that XL999 markedly inhibited the phosphorylation of FLT3-ITD and its key downstream effectors, including STAT5, extracellular signal-regulated kinase (ERK), and AKT, in both MV4-11 and MOLM-13 cells (Fig. 2b). Furthermore, cell cycle analysis revealed that 10 nM XL999 induced prominent cell cycle arrest at 24 h in FLT3-ITD-positive cells; conversely, concentrations up to 100 nM elicited no significant cell cycle disruption in FLT3-ITD-negative OCI-AML3 cells (Fig. 2c and Fig. S4a). Consistent with these findings, XL999 triggered dose-dependent apoptosis in MOLM-13 and MV4-11 cells following 48 h of treatment (Fig. 2d and Fig. S4b), concomitant with caspase-8 activation and PARP1 cleavage (Fig. 2e). In contrast, no appreciable proapoptotic activity was observed in OCI-AML3 cells even at 100 nM (Fig. 2d and Fig. S4b). Notably, the ability of XL999 to induce cell cycle arrest and apoptosis was superior to that of gilteritinib, which was consistent with its increased potency observed in the cell viability assays.

### XL999 exhibits feasible oral bioavailability and acceptable short-term tolerability

**Fig. 3:**
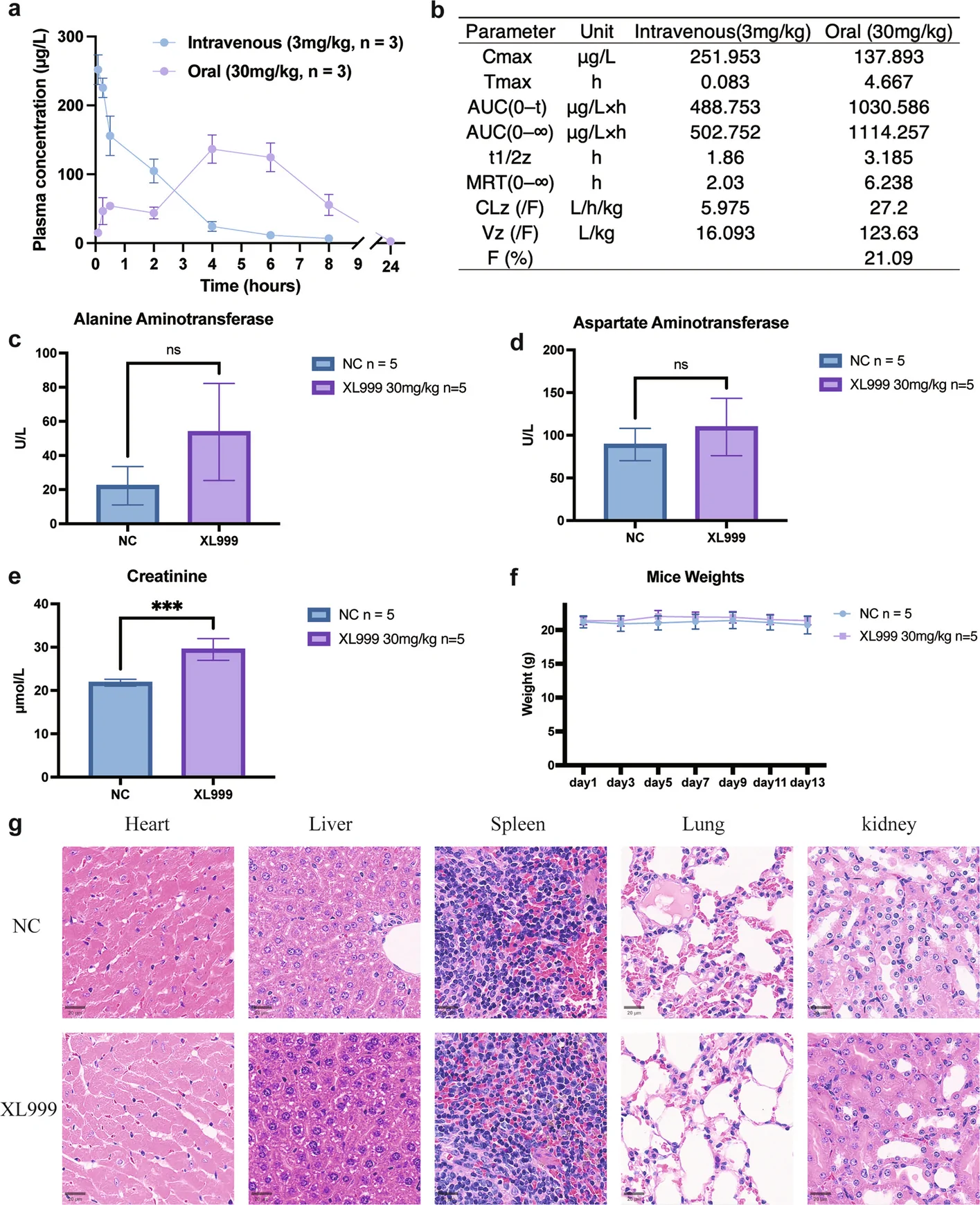
Pharmacokinetic characterization and preliminary in vivo tolerability evaluation of XL999. **a** Plasma concentration–time profiles of XL999 following a single intravenous (i.v., 3 mg/kg, *n = *3) or oral (p.o., 30 mg/kg, *n = *3) administration in Sprague–Dawley rats. **b** Summary of key pharmacokinetic parameters of XL999 following i.v. and p.o. administration in rats. Cmax, peak plasma concentration; Tmax, time to reach peak plasma concentration; AUC, area under the concentration–time curve; t1/2z, elimination half-life; MRT, mean residence time; CLz, systemic clearance; Vz, apparent volume of distribution; F, absolute oral bioavailability. **c**–**e** Serum biochemistry parameters including **c** alanine aminotransferase (ALT), **d** aspartate aminotransferase (AST), and **e** creatinine in BALB/c mice measured 48 h post-administration of vehicle control or XL999 (30 mg/kg, p.o.) (*n = *5 mice per group). **f** Longitudinal body weight monitoring of mice over 14 days of continuous daily oral treatment (*n = *5 mice per group). **g** Representative hematoxylin and eosin (H&E) staining of major organs (heart, liver, spleen, lung, and kidney) harvested on day 14 from vehicle- and XL999-treated mice, showing no overt treatment-related histopathological lesions within the observation timeframe (scale bars = 20 μm, *n = *5 mice per group). Data are presented as mean ± SD. ***P < 0.001; ns, not significant. Abbreviations: X, XL999; Q, quizartinib; G, gilteritinib; NC, vehicle control

To evaluate the pharmacokinetic (PK) properties and translational potential of XL999, a comparative PK study was conducted in Sprague–Dawley rats following a single intravenous (i.v., 3 mg/kg, *n = *3) or oral (p.o., 30 mg/kg, *n = *3) administration (Fig. 3a, b, Tables S1–S4).

Following i.v. administration, XL999 exhibited rapid systemic distribution and elimination, achieving a peak plasma concentration (Cmax) of 251.95 μg/L with an elimination half-life (t1/2z) of 1.86 h and total systemic exposure (AUC0–∞) of 502.75 μg/L·h (Fig. 3a, b). When administered orally, XL999 displayed smooth absorption, reaching a Cmax of 137.89 μg/L at Tmax = 4.67 h (Fig. 3a, b). Oral administration yielded an extended mean residence time (MRT0–∞ = 6.24 h vs. 2.03 h for i.v.) and an elimination half-life of 3.19 h, resulting in an AUC0–t of 1030.59 μg/L·h and an AUC0–∞ of 1114.26 μg/L·h (Fig. 3b). The calculated absolute oral bioavailability (F) was 21.09%, demonstrating feasible systemic absorption following oral administration in rats (Fig. 3b).

In parallel, the short-term in vivo tolerability and preliminary biosafety of XL999 were evaluated in BALB/c mice receiving daily oral administration (30 mg/kg/day, *n = *5) or vehicle control (NC, *n = *5) for 14 days (Fig. 3c–g). Serum biochemical analysis performed 48 h after the first oral administration revealed that alanine aminotransferase (ALT) and aspartate aminotransferase (AST) levels remained comparable between the two groups (Fig. 3c, d). Although serum creatinine (Cr) was modestly increased in XL999-treated mice compared with controls (29.48 vs. 21.80 μmol/L, *P < *0.001; Fig. 3e), the mean value remained within the physiological reference range. Continuous monitoring throughout the 14-day treatment period revealed no marked body weight loss, indicating acceptable tolerability at the evaluated oral dose (Fig. 3f). Furthermore, histopathological examination using hematoxylin and eosin (H&E) staining on day 14 showed no overt pathological abnormalities in the heart, liver, spleen, lung, or kidney (Fig. 3g). Overall, these findings suggested that orally administered XL999 exhibited an acceptable short-term tolerability profile under the evaluated dosing regimen.

### XL999 demonstrates a significant antitumor effect in the Ba/F3-FLT3-ITD mouse model

**Fig. 4:**
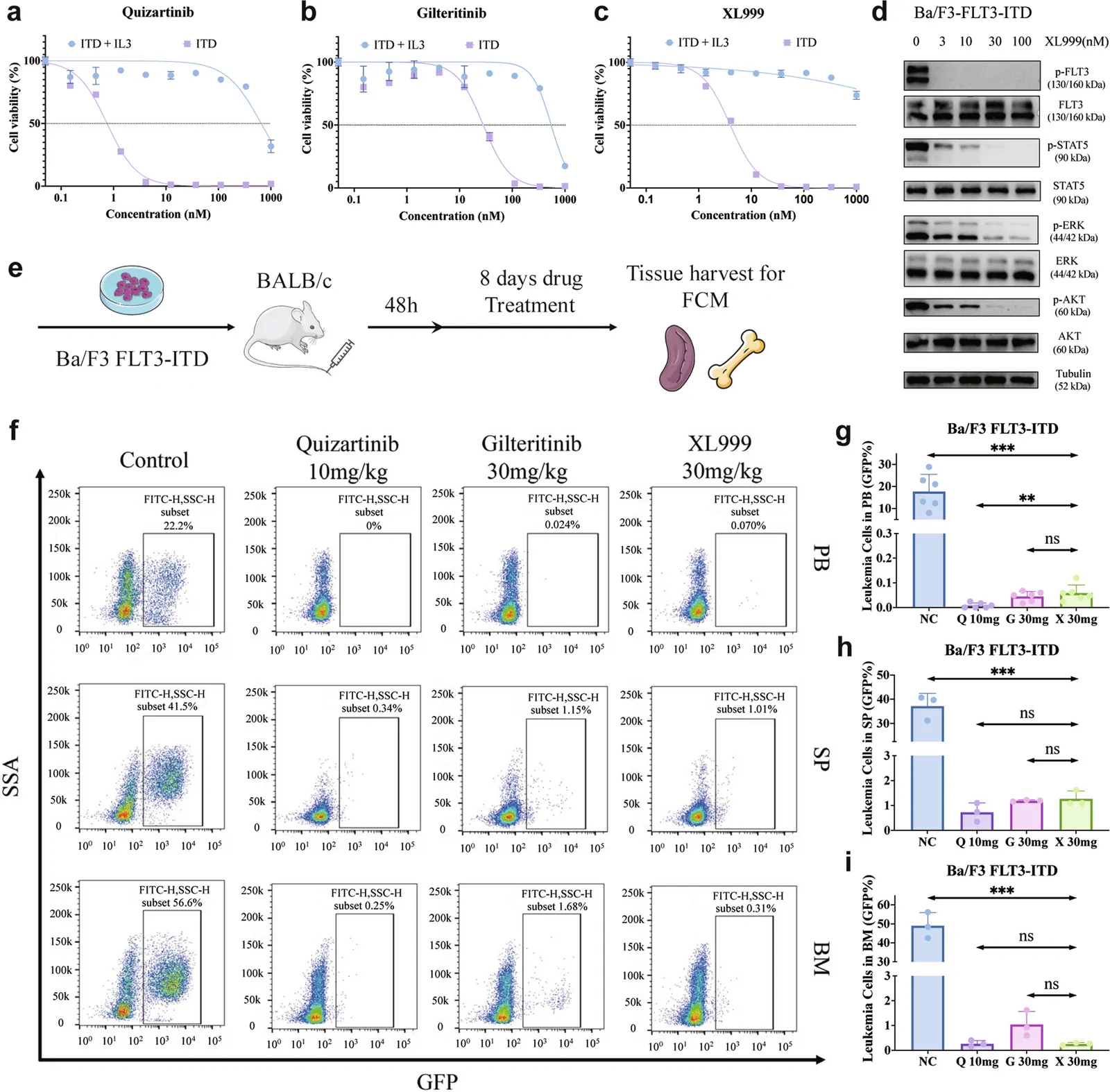
XL999 exhibits potent anti-leukemic activity against FLT3-ITD in vitro and in vivo. **a**–**c** Dose–response curves of Ba/F3-FLT3-ITD cells treated with quizartinib (**a**), gilteritinib (**b**), or XL999 (**c**) for 48 h, evaluated under FLT3-ITD conditions in the absence or presence of interleukin-3. **d** Immunoblot analysis of total and phosphorylated (p-) FLT3, STAT5, ERK, and AKT in Ba/F3-FLT3-ITD cells following treatment with increasing concentrations of XL999 (0–100 nM) for 6 h. Tubulin was used as the loading control. **e** Schematic representation of the leukemia mouse model established by intravenous injection of Ba/F3-FLT3-ITD cells into BALB/c mice, followed by a 48 h engraftment period and an 8-day treatment regimen. **f** Representative flow cytometry dot plots showing GFP-positive leukemia cell infiltration in peripheral blood (PB), spleen (SP), and bone marrow (BM) across the indicated treatment groups. **g**–**i** Quantification of GFP-positive leukemia cell burden in the **g** PB, **h** SP, and **i** BM (*n = *6 mice per group for PB; *n = *3 for SP and BM). Data are presented as mean ± SD. Statistical significance is indicated as **P < 0.01, ***P < 0.001, and ns, not significant. Abbreviations: X, XL999; Q, quizartinib; G, gilteritinib; NC, vehicle control

Consistent with its antiproliferative effects on MOLM-13 and MV4-11 cells, XL999 robustly suppressed the in vitro growth of Ba/F3-FLT3-ITD cells, with an IC_50_ value of 3.99 nM. This potency surpassed that of gilteritinib (IC_50_ = 28.14 nM), albeit remaining lower than that of quizartinib (IC_50_ = 0.75 nM) (Fig. 4a–c). Furthermore, compared with Ba/F3-FLT3-ITD cells, Ba/F3 cells overexpressing wild-type human FLT3 (supplemented with 100 nM FLT3 ligand) had an IC_50_ of 8.147 nM, reflecting lower sensitivity (Fig. S5a). Concurrently, western blot analysis verified that XL999 effectively inhibited the phosphorylation of FLT3 and its downstream signaling cascades in Ba/F3-FLT3-ITD cells (Fig. 4d).

We next investigated the in vivo antileukemic potential of XL999 using a Ba/F3-FLT3-ITD mouse model (Fig. 4e). The mice were intravenously inoculated with 5 × 10^5^ cells and allowed to stabilize for 48 h prior to receiving treatment via daily oral gavage for 8 days. We monitored the frequency of green fluorescent protein (GFP) positive cells across different anatomical compartments by flow cytometry. In the peripheral blood (PB), the percentage of mutant cells decreased to 0.059% upon XL999 administration; this residual frequency was marginally higher than that in the quizartinib cohort (0.0082%, *P* = 0.005) but statistically indistinguishable from that in the gilteritinib cohort (0.045%, *P* = 0.41; Fig. 4f, g). With respect to the splenic compartment, XL999-treated mice harbored only 1.27% GFP-positive cells in the spleen (SP), whereas malignant cells in the vehicle group expanded to 37.20% (*P < *0.001; Fig. 4f, h). Finally, a medullary evaluation revealed that the percentage of GFP-positive cells in the BM was profoundly reduced to 0.26% in the XL999 group, whereas it was 49.03% in the vehicle control group (*P < *0.001; Fig. 4f, i). Across both the splenic and the medullary compartments, the antileukemic effect of XL999 matched the effects of gilteritinib and quizartinib.

### XL999 overcomes secondary FLT3 resistance mutations in vitro

**Fig. 5:**
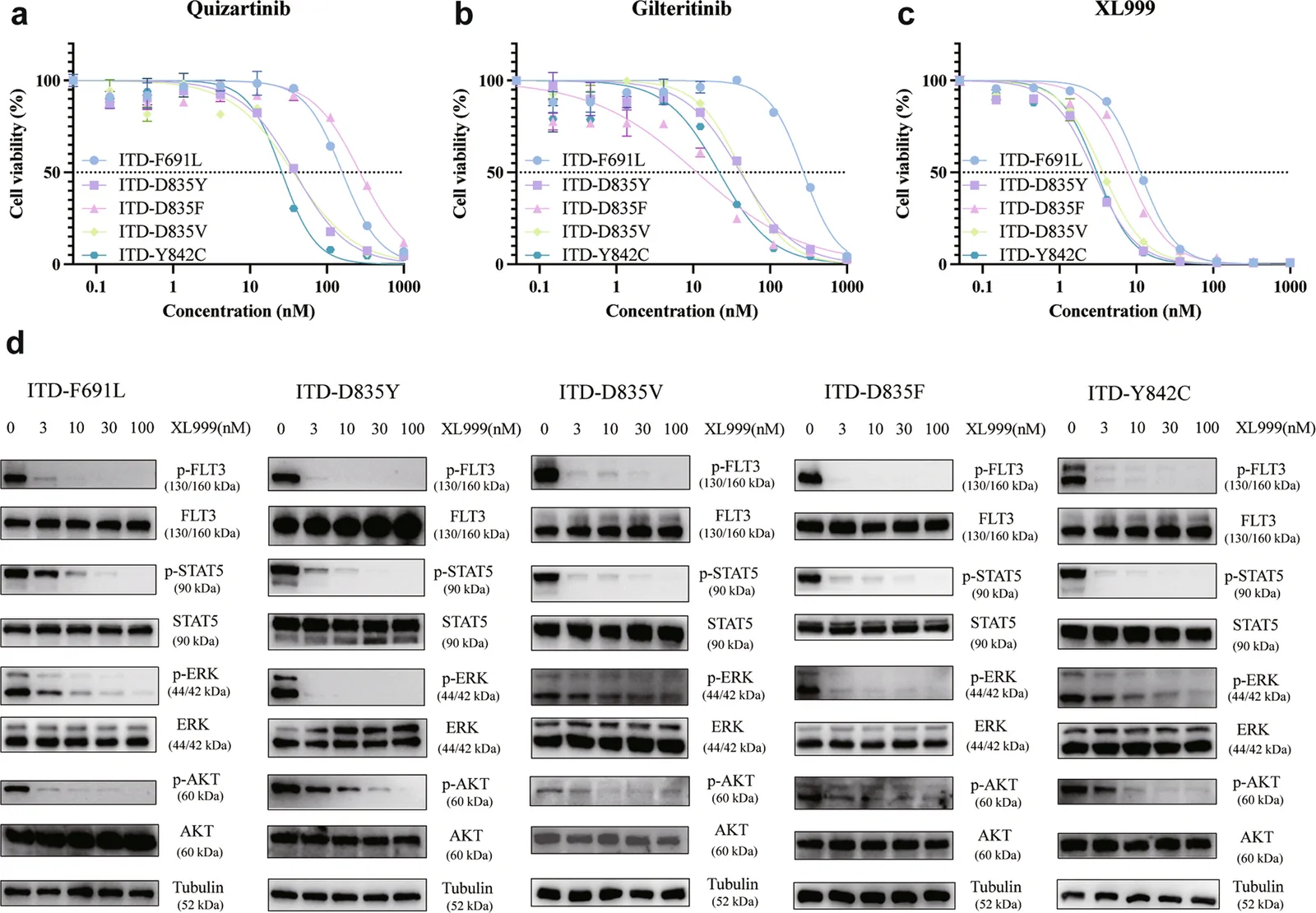
XL999 overcomes secondary FLT3-ITD tyrosine kinase domain resistance mutations in vitro. **a**–**c** Dose–response curves of Ba/F3 cells expressing FLT3-ITD harboring secondary tyrosine kinase domain (TKD) mutations, including F691L, D835Y, D835V, D835F, and Y842C, following 48 h of treatment with quizartinib (**a**), gilteritinib (**b**), or XL999 (**c**). Cell viability was normalized to vehicle controls, and the dashed horizontal line indicates 50% growth inhibition. Data are presented as mean ± SD from three independent experiments. **d** Immunoblot analysis of total and phosphorylated (p-) FLT3, STAT5, ERK, and AKT in Ba/F3-FLT3-ITD-TKD mutant cell lines treated with increasing concentrations of XL999 (0–100 nM) for 6 h. Tubulin was used as the loading control

To determine whether XL999 could circumvent secondary mutations conferring resistance to current FLT3 inhibitors, we generated a panel of Ba/F3 cell lines expressing FLT3-ITD in combination with secondary TKD mutations, including the F691L gatekeeper mutation. Proliferation assays confirmed that all secondary mutations conferred resistance to quizartinib (Fig. 5a), and gilteritinib retained efficacy only against variants other than the F691L mutant (Fig. 5b). Remarkably, XL999 potently suppressed the growth of cells harboring each of the tested secondary mutations, with IC_50_ values ranging from 2.81 to 11.23 nM (Fig. 5c). Consistent with these phenotypic results, XL999 effectively inhibited the phosphorylation of FLT3 and its downstream cascades (STAT5, ERK, and AKT) (Fig. 5d). We also evaluated the potency of XL999 against isolated, clinically relevant FLT3-TKD mutants using Ba/F3 cells harboring individual D835Y, D835V, or Y842C mutations (Fig. S5b–d). Compared with gilteritinib, XL999 displayed superior potency against D835Y (IC_50_: 0.48 nM vs. 0.68 nM) and Y842C (IC_50_: 0.12 nM vs. 0.60 nM) but maintained a comparable but marginally higher IC_50_ against D835V (0.49 nM vs. 0.31 nM). Collectively, these findings underscore that XL999 preserves robust activity against isolated FLT3-TKD mutations.

### XL999 demonstrates potent in vivo efficacy against secondary FLT3 resistance mutations

**Fig. 6:**
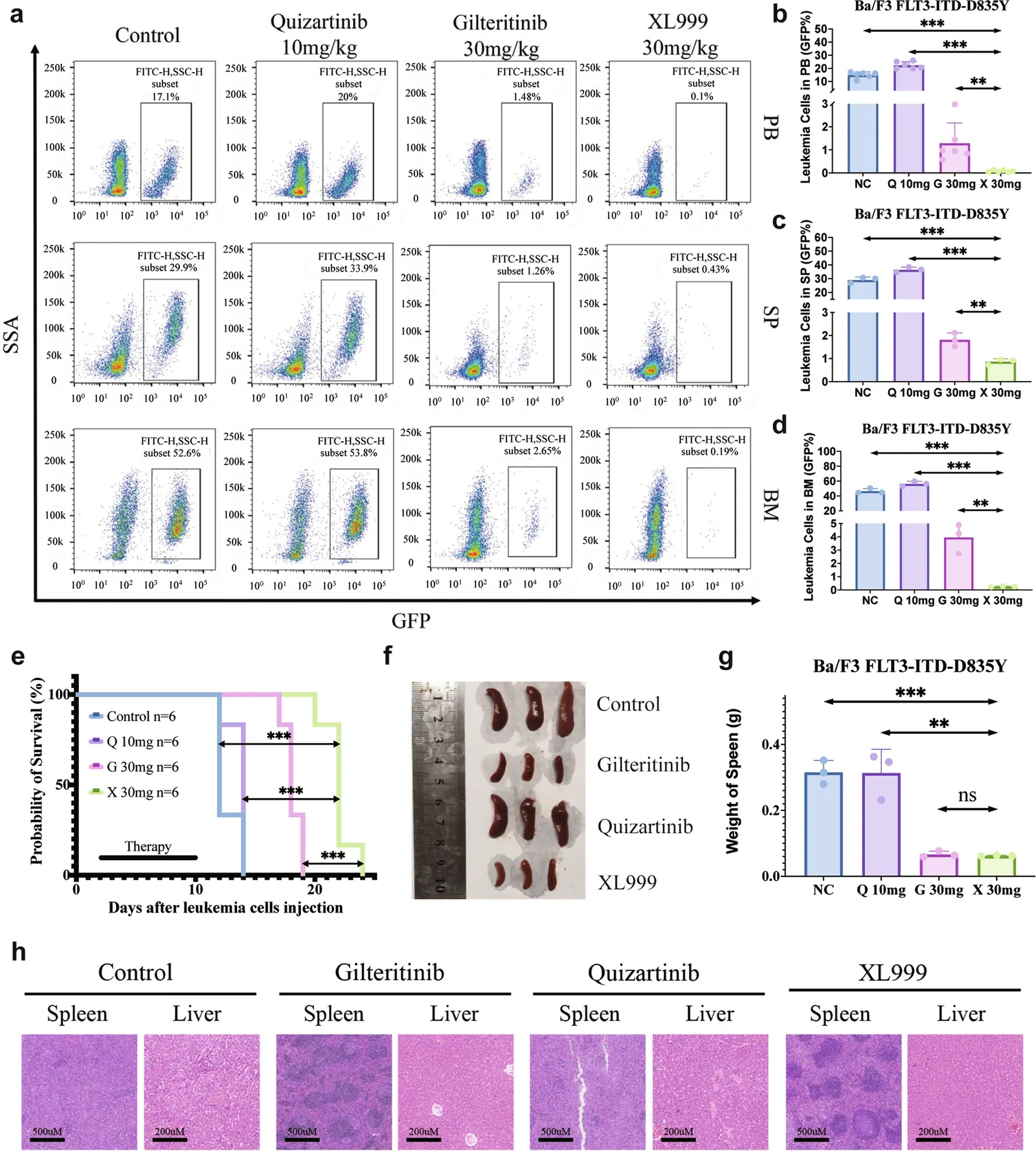
XL999 overcomes FLT3-ITD-D835Y–mediated resistance to type II FLT3 inhibitors in vivo. **a**–**d** Evaluation of leukemia burden in hematopoietic tissues of Ba/F3-FLT3-ITD-D835Y mice at day 12 post-transplantation (*n = *6 mice per group for peripheral blood (PB); *n = *3 for spleen (SP) and bone marrow (BM)). **a** Representative flow cytometry dot plots showing GFP-positive leukemia cell infiltration. **b**–**d** Quantification of GFP-positive leukemia cell percentages in the **b** PB, **c** SP, and **d** BM. The quizartinib group showed minimal therapeutic response, consistent with type II inhibitor resistance. **e** Kaplan–Meier survival analysis of Ba/F3-FLT3-ITD-D835Y-bearing mice treated daily with vehicle control (NC), quizartinib (10 mg/kg), gilteritinib (30 mg/kg), or XL999 (30 mg/kg) (*n = *6 mice per group). **f**–**g** Assessment of splenomegaly at day 12. **f** Representative images of harvested spleens and **g** quantitative analysis of spleen weights across treatment groups (*n = *3 mice per group). **h** Representative hematoxylin and eosin (H&E)–stained sections of spleen and liver tissues from each group (*n = *3 mice per group), demonstrating leukemic infiltration across organs. Scale bars, 500 μm and 200 μm as indicated. Data are presented as mean ± SD. Statistical significance is indicated as **P < 0.01, ***P < 0.001, and ns, not significant. Abbreviations: NC, vehicle control; Q, quizartinib; G, gilteritinib; X, XL999

To determine whether this broad-spectrum in vitro potency could be translated in vivo to overcome resistance driven by secondary mutations, an allograft leukemia model was established using Ba/F3 cells expressing the FLT3-ITD-D835Y mutation (Fig. S6a). As anticipated, the type II inhibitor quizartinib failed to produce a meaningful therapeutic response. Conversely, the type I inhibitor XL999 demonstrated prominent efficacy in curbing mutant cell accumulation. Flow cytometric analysis revealed that XL999 suppressed the percentage of GFP-positive cells in the PB to 0.073% (vs. 15.03% in the vehicle control, *P < *0.001), whereas gilteritinib-treated mice maintained a significantly greater residual load of 1.295% (*P* = 0.0064) (Fig. 6a, b). In the spleen, a robust reduction in the percentage of mutant cells to 0.87% was achieved by XL999 compared with 29.13% in the vehicle group (*P < *0.001), surpassing the efficacy of gilteritinib (1.82%, *P* = 0.0069) (Fig. 6a, c). Similarly, evaluation of the BM revealed that XL999 reduced the percentage of medullary GFP-positive cells to 0.25% (vs. 46.53% in the vehicle group, *P < *0.001), whereas gilteritinib left a substantial residual fraction of 3.96% (*P* = 0.0044) (Fig. 6a, d). This profound systemic clearance by XL999 translated into a substantial survival benefit. XL999 significantly extended the median overall survival to 22 days, compared with the vehicle control group that had a median overall survival of 12 days (*P < *0.001; Fig. 6e). Notably, the gilteritinib cohort exhibited a median overall survival of 18 days, over which XL999 achieved a statistically superior therapeutic advantage (*P < *0.001; Fig. 6e). Furthermore, tissue analysis revealed normalized spleen sizes (Fig. 6f, g), and H&E-stained sections confirmed intact structures without apparent abnormalities in both the spleen and liver (Fig. 6h and Fig. S6b).

**Fig. 7:**
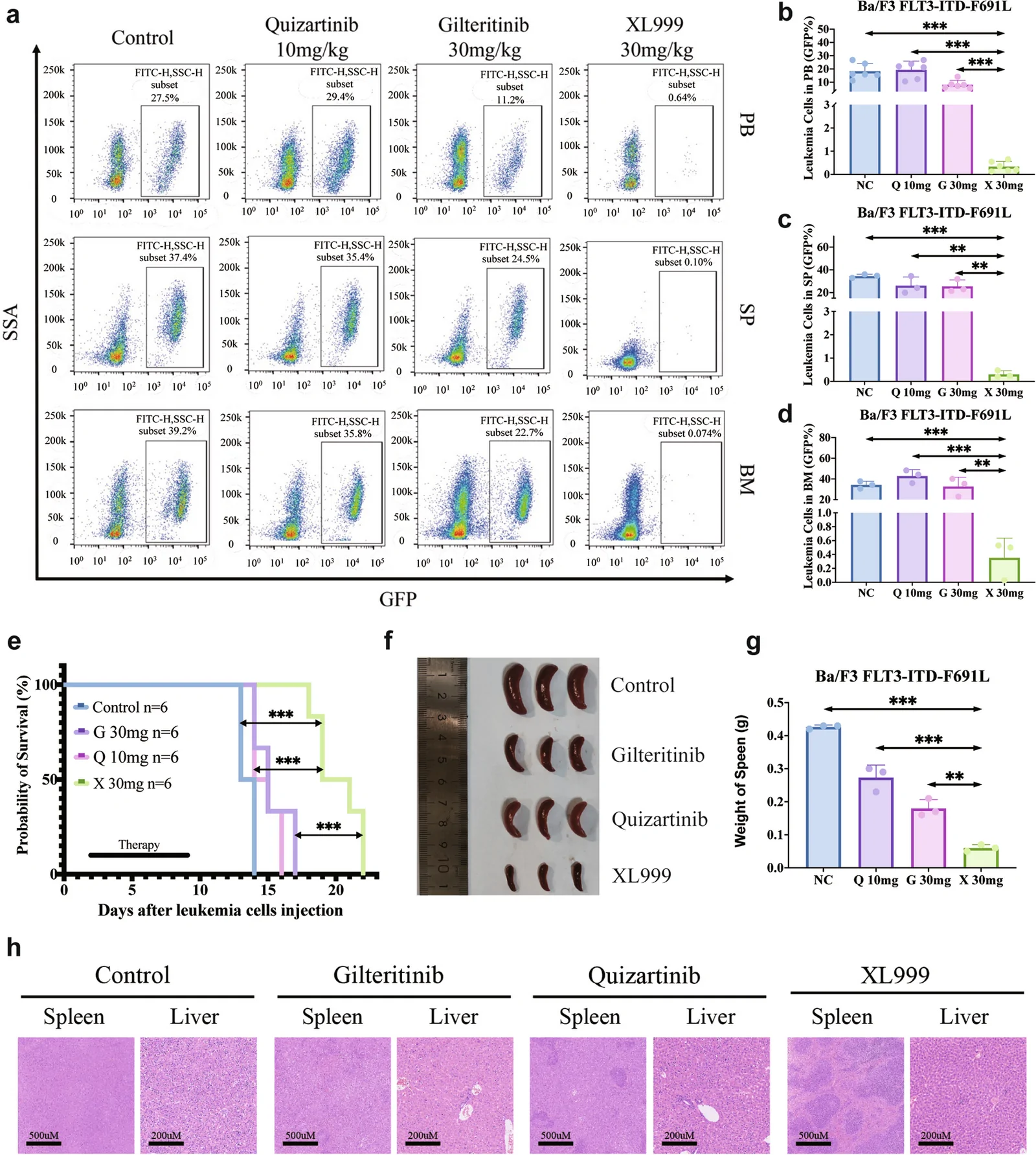
XL999 overcomes resistance mediated by the FLT3-ITD-F691L gatekeeper mutation in vivo. **a**–**d** Evaluation of leukemia burden in hematopoietic tissues of Ba/F3-FLT3-ITD-F691L mice at day 12 post-transplantation (*n = *6 mice per group for peripheral blood (PB); *n = *3 for spleen (SP) and bone marrow (BM)). **a** Representative flow cytometry dot plots showing GFP-positive leukemia cell infiltration across the indicated treatment groups. **b**–**d** Quantification of GFP-positive leukemia cell percentages in the **b** PB, **c** SP, and **d** BM. XL999 markedly reduced leukemic burden and effectively overcame gatekeeper mutation–mediated resistance compared with quizartinib and gilteritinib. **e** Kaplan–Meier survival analysis of Ba/F3-FLT3-ITD-F691L-bearing mice treated daily with vehicle control (NC), quizartinib (10 mg/kg), gilteritinib (30 mg/kg), or XL999 (30 mg/kg) (*n = *6 mice per group). **f**–**g** Assessment of splenomegaly at day 12. **f** Representative macroscopic images of harvested spleens and **g** quantitative analysis of spleen weights across treatment groups (*n = *3 mice per group). **h** Representative hematoxylin and eosin (H&E)–stained sections of spleen and liver tissues (*n = *3 mice per group), demonstrating reduced leukemic infiltration in the XL999-treated group. Scale bars, 500 μm and 200 μm as indicated. Data are presented as mean ± SD. Statistical significance is indicated as **P < 0.01, ***P < 0.001. Abbreviations: NC, vehicle control; Q, quizartinib; G, gilteritinib; X, XL999

Structurally, gatekeeper resistance mediated by the F691L mutation presents a major challenge because both gilteritinib and quizartinib depend on interactions with the F691 residue [41, 42]. Considering that molecular modeling predicted that XL999 binds independently of the F691 locus, we challenged a Ba/F3-FLT3-ITD-F691L mouse model to validate this structural rationale in vivo (Fig. S7a). Consistent with our predictions, quizartinib yielded no detectable antileukemic activity, whereas XL999 effectively eliminated the gatekeeper-mutant cells. In the PB, XL999 limited the mutant cell fraction to 0.34%, whereas the vehicle group showed a high accumulation of 18.32%, and gilteritinib resulted in a substantial residual frequency of 8.29% (*P < *0.001; Fig. 7a, b). A similar pattern was observed in the spleen, where the percentage of GFP-positive cells was only 0.31% in the XL999 cohort versus 34.57% in the vehicle control group (*P < *0.001) and 25.43% in the gilteritinib group (*P* = 0.0015) (Fig. 7a, c). Medullary analysis further revealed that the percentage of GFP-positive cells in the BM decreased to 0.35% in the XL999 treatment group compared with 34.23% in the vehicle group (*P < *0.001) and 32.77% in the gilteritinib cohort (*P* = 0.0032) (Fig. 7a, d). Consequently, XL999 extended the median survival to 20 days, compared with the vehicle group which had a median survival of 13.5 days, and the gilteritinib cohort with a median survival of 15 days (both *P < *0.001; Fig. 7e). These results were supported by the notable normalization of the spleen weight in the XL999 group (Fig. 7f, g). Histopathology via H&E staining revealed that XL999 shielded the spleen and liver tissues from the severe blast infiltration and structural breakdown observed in the other groups (Fig. 7h, Fig. S7b).

### XL999 demonstrates superior efficacy in primary patient samples and a patient-derived xenograft model

**Fig. 8:**
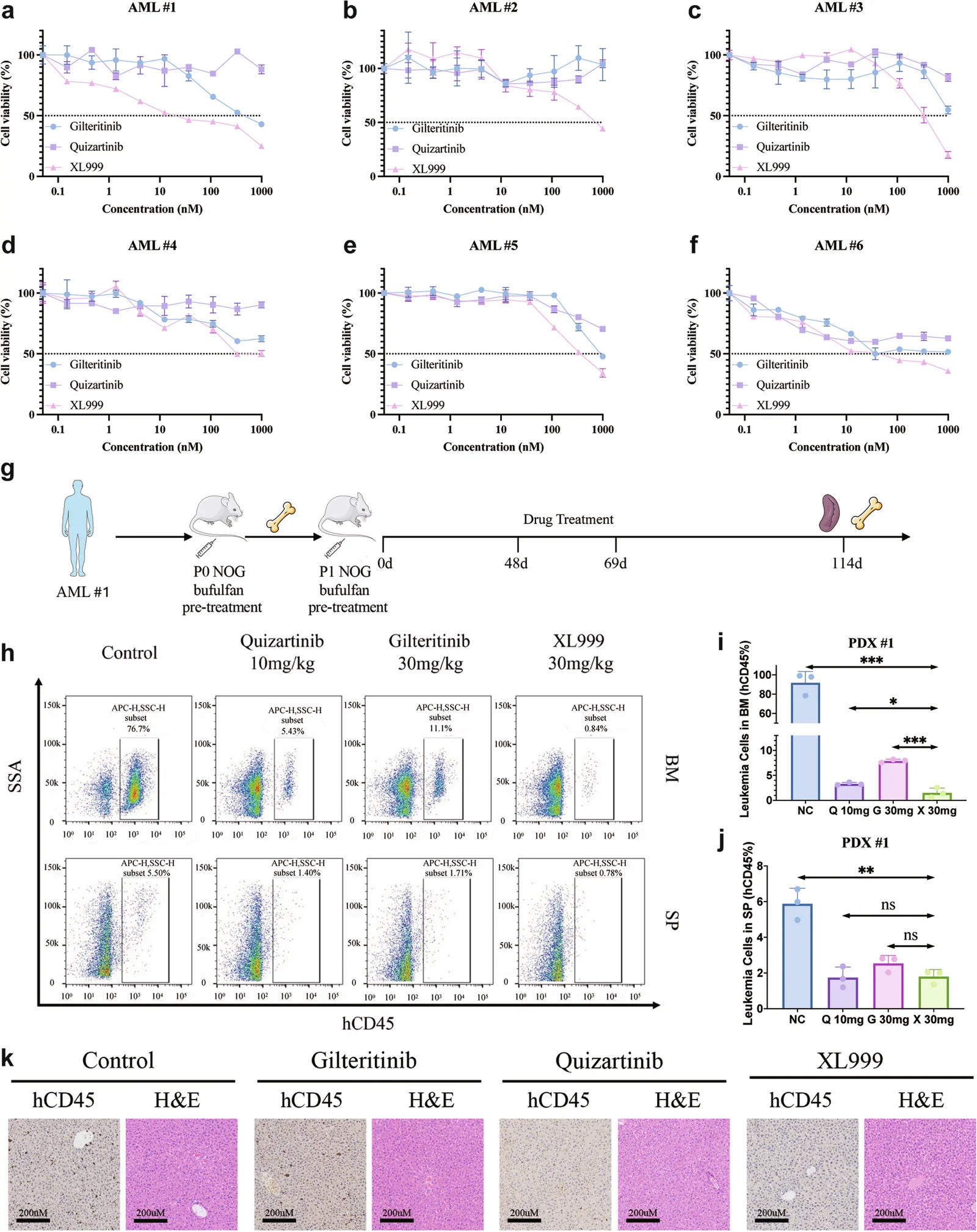
XL999 shows potent antileukemic activity against primary AML blasts ex vivo and suppresses leukemia progression in a patient-derived xenograft (PDX) model. **a**–**f** Ex vivo dose–response curves of primary human AML blasts isolated from patients harboring FLT3-ITD mutations. Cells were treated with increasing concentrations of XL999, gilteritinib, or quizartinib for 48 h prior to viability assessment. **g** Schematic workflow of the patient-derived xenograft (PDX) model establishment and treatment schedule. C-NKG mice were preconditioned with busulfan prior to intravenous transplantation of primary AML cells. **h** Representative flow cytometry dot plots showing human CD45⁺ (hCD45⁺) leukemic cell infiltration in bone marrow (BM) and spleen (SP) at day 114. **i**–**j** Quantification of hCD45⁺ leukemia cell burden in the **i** BM and **j** SP (*n = *3 mice per group). **k** Representative hematoxylin and eosin (H&E) staining and immunohistochemical (IHC) detection of hCD45 in liver tissue sections from PDX mice across the indicated treatment groups (*n = *3 mice per group). Scale bars, 200 μm. Data are presented as mean ± SD. Statistical significance is indicated as *P < 0.05, **P < 0.01, ***P < 0.001, and ns, not significant. Abbreviations: NC, vehicle control; Q, quizartinib; G, gilteritinib; X, XL999

To evaluate the preclinical efficacy of XL999 in patient-derived primary samples, BM mononuclear cells were isolated from nine patients with FLT3-ITD-mutant AML (whose clinical information is presented in Table S5). Ex vivo cell viability assays confirmed that XL999 dose-dependently suppressed primary blast growth and displayed comparable or superior potency to both gilteritinib and quizartinib (Fig. 8a–f and Fig. S8a–c). To further determine the translational potential of XL999, we established a patient-derived xenograft (PDX) model utilizing primary cells from a patient with AML (designated Patient #1; Fig. 8g). Following the therapeutic regimen, human cell chimerism was evaluated across distinct anatomical compartments. In the BM, the frequency of human CD45-positive (hCD45^+^) cells was profoundly suppressed to 1.49% under XL999 treatment, representing a striking reduction from the 91.77% observed in the vehicle group (*P < *0.001; Fig. 8h, i). Notably, this medullary clearance by XL999 was superior to that of gilteritinib (7.91%, *P < *0.001) and quizartinib (3.30%, *P* = 0.035). With respect to the splenic compartment, the proportion of hCD45^+^ cells decreased to 1.80% in the XL999 group compared with 5.88% in the vehicle control group (*P* = 0.0018; Fig. 8h, j), although no statistically significant differences were observed among the three inhibitor-treated cohorts within this organ. Finally, immunohistochemical (IHC) analysis of liver sections revealed that the density of infiltrating hCD45^+^ cells was markedly lower in the XL999 group than in either the gilteritinib or quizartinib cohort (Fig. 8k and Fig. S8d).

Collectively, these findings provide compelling evidence that XL999 outperforms current FLT3 inhibitors in markedly reducing leukemic burden both in vitro and in vivo.

## Discussion

AML is a highly heterogeneous malignancy driven by diverse chromosomal abnormalities and genetic mutations. Activating mutations in FLT3, particularly the FLT3-ITD mutation, which occurs in approximately 25% of patients, represent critical molecular drivers strongly associated with an adverse prognosis. Although second-generation FLT3 inhibitors, such as gilteritinib and quizartinib, have significantly advanced the therapeutic landscape, drug resistance and subsequent disease relapse remain virtually inevitable. Selective drug pressure frequently drives the emergence of on-target secondary mutations within the tyrosine kinase domain, most notably at residues D835 and Y842 and at the “gatekeeper” position, F691. The type I inhibitor gilteritinib can counteract certain secondary mutations (e.g., D835Y); however, no approved targeted therapies currently exist to effectively overcome the clinically challenging F691L gatekeeper mutation, leaving a substantial unmet medical need in the management of resistant, FLT3-driven AML.

To address this clinical vulnerability, we screened a kinase inhibitor library and identified XL999, a multitargeted, spectrum-selective kinase inhibitor, as a potent lead candidate with activity against clinically relevant FLT3 resistance mutations. In silico modeling provided a plausible structural hypothesis, suggesting that XL999 acted as a type I FLT3 inhibitor and sustained its binding through a distributed hydrogen-bonding network potentially independent of the canonical F691 gatekeeper residue. Direct target engagement was demonstrated by CETSA in Ba/F3-FLT3-ITD cells, coupled with a remarkably low IC_50_ value (4.16 nM) in an ADP-Glo assay using purified recombinant FLT3-ITD oncoproteins. We further evaluated the specificity and efficacy of XL999 across a panel of leukemia cell lines and demonstrated its selective antileukemic activity in vitro, which was subsequently validated in vivo using a Ba/F3-FLT3-ITD mouse model. In mice harboring Ba/F3-FLT3-ITD-D835Y or -F691L resistance mutations, compared with gilteritinib and quizartinib, XL999 demonstrated greater antileukemic activity and significantly prolonged survival. Finally, the enhanced antileukemic activity of XL999 relative to that of clinically available FLT3 inhibitors was further supported in primary patient-derived cells and a PDX model.

Importantly, our study provides the first systematic evaluation of the oral pharmacokinetics and preliminary biosafety of XL999, helping to address an important knowledge gap left by its previous clinical development. We demonstrated that XL999 exhibited an absolute oral bioavailability of 21.09% in rats, with a prolonged absorption phase, supporting its potential for oral administration. Furthermore, a two-week oral tolerability assessment in mice at 30 mg/kg suggested an acceptable short-term tolerability profile, as evidenced by stable body weight, comparable ALT and AST levels, serum creatinine remaining within the physiological reference range, and no overt histopathological abnormalities in major organs. In previous phase II clinical trials, a weekly intravenous dose of 2.4 mg/kg XL999 was associated with several adverse effects, including fatigue, hypertension, nausea, and serious cardiac adverse events (SAEs), which potentially stemmed from its potent inhibition of VEGFR2 and FGFR1 [43, 44]. Although those clinical trials were prematurely terminated because of cardiotoxicity, these cardiac SAEs typically occurred after the first dose and were largely reversible [38]. Notably, whereas the historical intravenous regimen reached a Cmax of 519 µg/L in humans, our oral regimen (30 mg/kg) achieved robust antileukemic efficacy at a substantially reduced Cmax of only 137.89 µg/L. The reduced peak exposure observed after oral administration may have contributed to the acceptable short-term tolerability observed in our preclinical study. However, direct comparisons between historical clinical intravenous exposure and our preclinical oral pharmacokinetic data should be interpreted with caution because of interspecies differences, administration routes, and dosing schedules.

In addition to on-target secondary point mutations, resistance to FLT3 inhibitors frequently involves microenvironment-mediated and off-target escape mechanisms. For instance, elevated concentrations of FLT3 ligand within the BM microenvironment can sustain downstream MAPK pathway signaling, promoting the survival of leukemic blasts despite active FLT3 tyrosine kinase inhibitor (TKI). Furthermore, accelerated drug metabolism driven by CYP3A4 enzymes expressed in BM stromal cells can facilitate rapid drug clearance, fostering a protective niche that contributes to clinical resistance. In addition to genetic alterations driving persistent tyrosine kinase activation, the dysregulation of alternative kinases significantly contributes to the pathogenesis, progression, and therapeutic evasion of leukemia. For example, AXL is frequently overexpressed in patients with AML and directly participates in resistance to FLT3 inhibitors such as quizartinib and midostaurin [45–47]. Unlike quizartinib, which is a highly selective FLT3 inhibitor with minimal off-target activity, gilteritinib also targets AXL [48, 49]. This mechanistic difference may explain why quizartinib, despite demonstrating robust efficacy in in vitro cell lines and Ba/F3 mouse models, was inferior to gilteritinib monotherapy in a phase II clinical trial [50, 51]. Within this challenging landscape, the multitarget profile of XL999 offers a distinct advantage because of its ability to simultaneously block bypass pathways (including RET, SRC, and AXL) alongside its potent type I FLT3 inhibition. RET is critical across multiple AML subtypes and is frequently coexpressed with FLT3; its inhibition suppresses the growth of FLT3-dependent AML cells, accompanied by increased autophagy and FLT3 depletion [52]. Moreover, SRC serves as a key downstream mediator of FLT3-ITD that drives STAT5 activation [53]. Consequently, the ability of XL999 to concurrently inhibit AXL, RET, and SRC likely enhances its antileukemic potency, potentially accounting for its superior performance in primary patient-derived cells and PDX models.

Several limitations of this study should be acknowledged. First, although XL999 exhibited an acceptable short-term tolerability profile in mouse models, comprehensive cardiovascular safety evaluations, including electrocardiographic monitoring, cardiac biomarkers, and long-term cardiac toxicity assessments, were not performed. Therefore, whether oral administration of XL999 is associated with a reduced risk of cardiovascular toxicity associated with previous intravenous exposure remains to be determined. Second, pharmacokinetic comparisons between our preclinical oral administration study and historical clinical intravenous exposure should be interpreted with caution because of differences in species, dosing regimens, and pharmacokinetic characteristics. Third, although this study demonstrated potent activity against major FLT3 kinase domain-mediated resistance mutations, including D835Y and F691L, other noncanonical resistance mechanisms, such as RAS pathway activation, microenvironment-mediated protection, FLT3 ligand-driven signaling, CYP3A4-mediated drug metabolism, and clonal evolution, were not systematically evaluated. Future studies using clinically relevant resistance models and longitudinal molecular profiling are needed to further define the therapeutic potential of XL999.

In conclusion, our study suggests that XL999 functions as an orally bioavailable type I FLT3 inhibitor with activity against major clinically relevant FLT3 resistance mutations. These findings provide preclinical evidence supporting the further investigation of orally administered XL999 as a potential therapeutic candidate for patients with relapsed or refractory FLT3-ITD-positive AML.

## Materials and methods

### Reagents and cell culture

XL999, gilteritinib, and quizartinib were obtained from TargetMol (Boston, MA, USA), prepared as 10 mM DMSO stocks, and stored at −20 °C for in vitro studies. For in vivo administration, gilteritinib was formulated in 5% DMSO/35% PEG300/10% Tween 80/50% sterile water, quizartinib in 0.5% methylcellulose, and XL999 in 5% DMSO/40% PEG300/5% Tween 80/50% sterile water. The Protein Kinase Compound Library (HY-L196; MedChemExpress, USA) contained 2,807 small molecules.

Human leukemia cell lines (MOLM-13, MV4-11, RS4;11, KASUMI-1, U-937, HL-60, OCI-AML3, THP-1, HEL, and REH) were obtained from DSMZ or ATCC. Ba/F3 cells stably expressing human FLT3 or its mutants were generated as described previously [32]. Cells were cultured in RPMI-1640 containing 10% FBS and 1% penicillin–streptomycin at 37 °C with 5% CO₂ and routinely screened for mycoplasma using MycoBlue (Vazyme); detected contamination was eliminated with Myco-Off (InvivoGen).

### Kinase inhibitor library screening and validation

An unbiased subset of 400 consecutive compounds from the 2,807-member library was screened in Ba/F3-FLT3-ITD-F691L cells with or without IL-3. Viability after 48 h was measured using CellTiter-Glo® (Promega) and analyzed by hierarchical clustering (Fig. S1). Hits were validated in Ba/F3-FLT3-ITD cells (with or without IL-3) and Ba/F3-FLT3-ITD-D835Y/F691L cells (Fig. S2a).

### Immunoblotting

Cells were washed with cold PBS and lysed in RIPA buffer containing protease and phosphatase inhibitors. Proteins were separated by SDS-PAGE, transferred to nitrocellulose membranes, blocked in 5% non-fat milk/TBST for 1 h, and incubated overnight at 4 °C with primary antibodies in 5% BSA/TBST. Antibodies were against phospho-FLT3 (Tyr591; CST #3464), FLT3 (CST #3462), phospho-STAT5 (Tyr694; CST #9351), STAT5 (CST #9363), phospho-ERK1/2 (Thr202/Tyr204; CST #4370), ERK1/2 (CST #4695), phospho-AKT (Ser473; CST #4060), AKT (CST #9272), PARP1 (Proteintech 13,371–1-AP), and caspase-8 (Proteintech 13,423–1-AP). After washing, membranes were incubated for 1 h with HRP-conjugated anti-tubulin (Proteintech HRP-66031). Signals were detected by ECL on a ChemiDoc MP system (Bio-Rad) and quantified using ImageJ v1.5.

### Cellular thermal shift assay

Ba/F3-FLT3-ITD cells (1 × 10⁷) were treated with 1000 nM XL999 or 0.1% DMSO (vehicle control) for 1 h. Cells were then harvested, washed twice with cold PBS, and resuspended in PBS containing protease inhibitors (TargetMol, USA). The cell suspension was divided into six equal aliquots and heated at temperatures ranging from 37 to 54 °C for 3 min, followed by cooling to room temperature. Cell lysis was performed by three freeze–thaw cycles in liquid nitrogen. After centrifugation at 20,000 × g for 20 min at 4 °C, the soluble protein fraction in the supernatant was collected and retained. FLT3 expression levels were then evaluated by Western blot analysis.

### Molecular docking

The XL999 structure from PubChem was prepared in Schrödinger LigPrep using the OPLS_4 force field and Epik to generate relevant stereoisomers, tautomers, and protonation states. The FLT3 crystal structure (PDB 6JQR) was prepared with Protein Preparation Wizard by correcting bond orders, adding hydrogens, reconstructing missing side chains, removing water/cofactors, optimizing hydrogen bonds, and performing restrained OPLS_4 minimization. Docking was performed with Glide using the co-crystallized ligand-defined site (10 Å inner grid; outer box adjusted to ligand dimensions) in standard-precision mode, and poses were ranked by GlideScore.

### In vitro kinase panel inhibition assays

XL999 was screened at 100 nM against a 76-target tyrosine kinase panel (ICE Bioscience) using HTRF or ADP-Glo assays in 384-well plates. XL999 was dispensed using an Echo 655 (50 nL for HTRF; 40 nL for ADP-Glo), mixed with 2 × kinase and ATP/substrate solutions, and incubated at 25 °C for 60 min. For HTRF, XL665/antibody solution was added and incubated for 60 min before measuring the 665/620-nm fluorescence ratio. For ADP-Glo, ADP-Glo and kinase detection reagents were sequentially added with 40-min incubations. Signals were measured on a PHERAstar reader, and inhibition was calculated relative to 1% DMSO vehicle and 10 μM positive-control inhibitor.

### Cell viability assay

Cells were seeded in 96-well plates at densities ranging from 1,000 to 5,000 cells per well and treated with serial dilutions of compounds (final concentrations ranging from 0.15 to 1,000 nM). After 48 h of incubation, cell viability was assessed by measuring intracellular ATP levels using the CellTiter-Glo® Luminescent Cell Viability Assay (Promega, USA) according to the manufacturer’s instructions. Luminescence signals were measured using a Varioskan Flash microplate reader (Thermo Fisher Scientific, Waltham, MA, USA).

### Flow cytometry and analysis

MOLM-13 and MV4-11 cells were seeded in 6-well plates at a density of 2 × 10⁶ cells per well and treated with XL999 or gilteritinib for 24 h. Thereafter, cells were harvested, fixed in ice-cold 70% ethanol overnight at 4 °C, and resuspended in a staining solution containing RNase A (100 μg/mL) and propidium iodide (PI, 50 μg/mL). DNA content was analyzed by flow cytometry for cell cycle distribution analysis. For apoptosis analysis, cells were plated at 2 × 10^5^ cells per well and exposed to compounds for 48 h. Apoptosis was evaluated using the Dead Cell Apoptosis Kit (Invitrogen, Cat# BMS500FI, USA) according to the manufacturer’s instructions. For both assays, stained cells were analyzed using a BD LSRFortessa flow cytometer, and data were processed using FlowJo software (version 10).

### Patient primary samples

BM samples were obtained from FLT3-ITD-positive AML patients irrespective of age, sex, or disease status. BMMCs were isolated by Lymphoprep density-gradient centrifugation and cultured in RPMI-1640 with 20% FBS at 37 °C and 5% CO₂. Patient characteristics are provided in Supplementary Table S5. The study was approved by the Ethics Committee of Guangzhou First People's Hospital (K-2026–124-01) and conducted in accordance with the Declaration of Helsinki.

### Pharmacokinetic and bioavailability study

Male Sprague–Dawley rats (*n = *3/group) received a single intravenous (3 mg/kg) or oral (30 mg/kg) dose of XL999, with plasma collected through 48 h. Plasma (5 μL) was precipitated with 50 μL acetonitrile containing verapamil (10 ng/mL) as internal standard and analyzed by UFLC-MS/MS (QTRAP 5500, BEH C18) in positive MRM mode. Transitions were m/z 446.2 → 112.1 for XL999 (CE 34 eV) and 455.5 → 303.3 for verapamil (CE 37 eV). Pharmacokinetic parameters and absolute bioavailability were calculated by non-compartmental analysis using DAS 2.0.

### In vivo safety evaluation of XL999

Eight-week-old female BALB/c mice received XL999 (30 mg/kg, *n = *5) or sterile water (*n = *5) daily for 14 days. AST, ALT, and Cr were measured 48 h after the first dose, and body weight was recorded every two days. On day 14, mice were euthanized and the liver, spleen, heart, kidney, and lung were collected for H&E examination.

### Leukemia mouse models

Eight-week-old female BALB/c mice were intravenously injected with 5 × 10^5^ GFP-labeled Ba/F3 cells expressing FLT3-ITD, FLT3-ITD-D835Y, or FLT3-ITD-F691L. After 48 h, mice were randomized to XL999 (30 mg/kg), gilteritinib (30 mg/kg), quizartinib (10 mg/kg), or vehicle (*n = *9/group) by daily oral gavage for 8 days. When controls became moribund (days 11–13), three mice/group were euthanized for BM, spleen, and liver collection; the remaining six were used for retro-orbital blood collection and survival analysis. GFP-positive cells in PB, spleen, and BM were quantified by flow cytometry. Spleen weights were recorded, and spleen/liver tissues were fixed in 4% paraformaldehyde, paraffin-embedded, sectioned at 4 μm, and stained with H&E.

For the PDX model, eight-week-old female C-NKG mice (Cyagen) were preconditioned with busulfan (20 mg/kg, intraperitoneally) 24 h before intravenous transplantation of 2 × 10⁶ BMMCs from Patient #1. PB hCD45⁺ cells were monitored monthly by flow cytometry. BM cells from first-generation mice were pooled after chimerism was detected and transplanted into second-generation recipients (2 × 10⁶ cells/mouse), which were randomized to the four treatment groups above. After hCD45⁺ cells became detectable by weekly PB monitoring, treatment was administered for 3 weeks. At endpoint, hCD45⁺ frequencies in BM and spleen were measured by flow cytometry; liver sections underwent H&E and hCD45 IHC staining.

All animal procedures were approved by the Animal Ethics Committee of the Greater Bay Area Institute of Precision Medicine (Guangzhou) (IPMGBA-26–0014).

### Statistical analysis

Data were analyzed using GraphPad Prism 10 and R 4.6.0. Two-group comparisons used two-tailed unpaired Student’s t-tests, and survival was analyzed by Kaplan–Meier curves with log-rank tests. *P < *0.05 was considered significant (*P < 0.05, **P < 0.01, ***P < 0.001).

## Supplementary Information

Supplementary Material 1.

## Data Availability

All data that support the findings of this study are included in the article and its supplementary information. Additional requests should be addressed to the corresponding author.
