# Supplementary material for: The type I FLT3 inhibitor XL999 overrides secondary FLT3-ITD mutations that arise under gilteritinib and quizartinib treatment in acute myeloid leukemia
Source: Mol Biomed. 2026 Sep 4;7:161. doi: 10.1186/s43556-026-00561-x (PMC13545197; doi:10.1186/s43556-026-00561-x)
Supplement: Supplementary file 1 [file 43556_2026_561_MOESM1_ESM.pdf]

Article title:

The type I FLT3 inhibitor XL999 overrides secondary FLT3-ITD mutations that arise under gilteritinib and quizartinib treatment in acute myeloid leukemia.

Author names, Affiliations and Correspondence:

Shiyang Wang[1,#], Yvyin Zhang[2,#], Zhiwei Zhong[1,#], Jiajun He[3], Jiaqi Fan[1], Qi Xiang[1], Fangfang Yang[1], Yuping Zhang[1*], Shunqing Wang[1*] and Peihong Wang[1*]

1. Department of Hematology, Guangzhou First People’s Hospital, School of Medicine, South China University of Technology, Guangzhou, P.R. China.

2. Department of Hematology, Guangdong Provincial People's Hospital, School of Medicine, South China University of Technology, Guangzhou, P.R. China.

3. Department of Hematologic Oncology, Sun Yat-sen University Cancer Center, Guangzhou, P.R. China.

* Corresponding author:

Peihong Wang, Guangzhou First People’s Hospital, School of Medicine, South China University of Technology, Guangzhou 510000, P.R. China. E-mail: wangph91@163.com; Shunqing Wang, eywangshq@scut.edu.cn; and Yuping Zhang, jnzhangyuping@163.com.

# These authors contributed equally to this work.

Keywords:

XL999, FLT3-ITD, Acute myeloid leukemia, FLT3 resistance mutation, Gilteritinib, Quizartinib

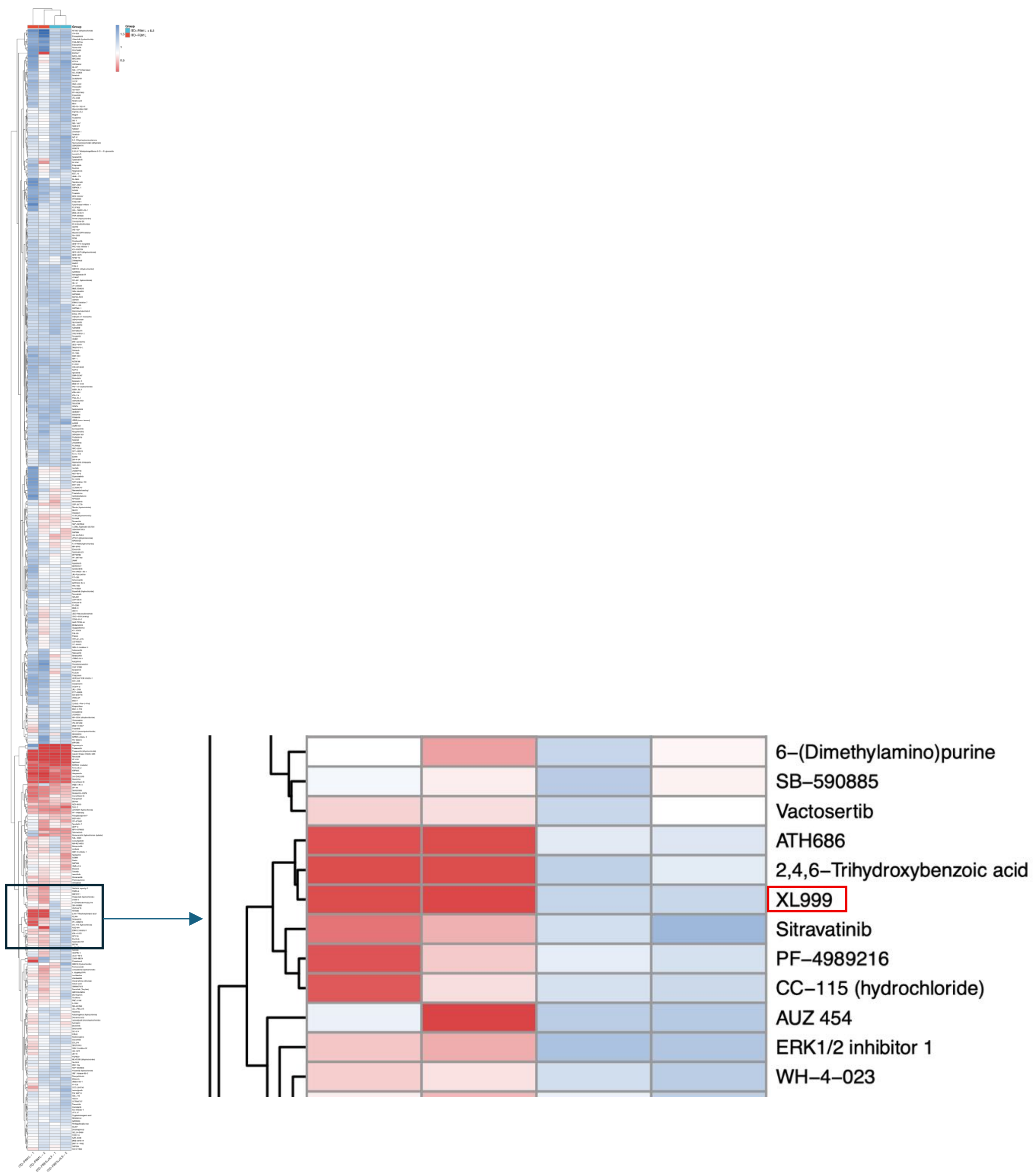

Supplementary Figure 1. High-throughput drug screening and activity-based clustering in Ba/F3-FLT3-ITD-F691L cells. Cells were treated for 48 h with 400 selected compounds from a 2,807-member kinase inhibitor library, either in the absence (F691L-1, F691L-2) or presence of interleukin-3 (F691L+IL-3-1, F691L+IL-3-2). Relative cell viability is indicated by the color gradient as shown in the scale bar. Compounds including ATH686, 2,4,6-trihydroxybenzoic acid, XL999, and sitravatinib were grouped based on similar inhibitory activity profiles against Ba/F3-FLT3-ITD-F691L cells. Notably, the cytotoxic effects of these compounds were fully rescued by interleukin-3 supplementation, indicating FLT3-dependent activity.

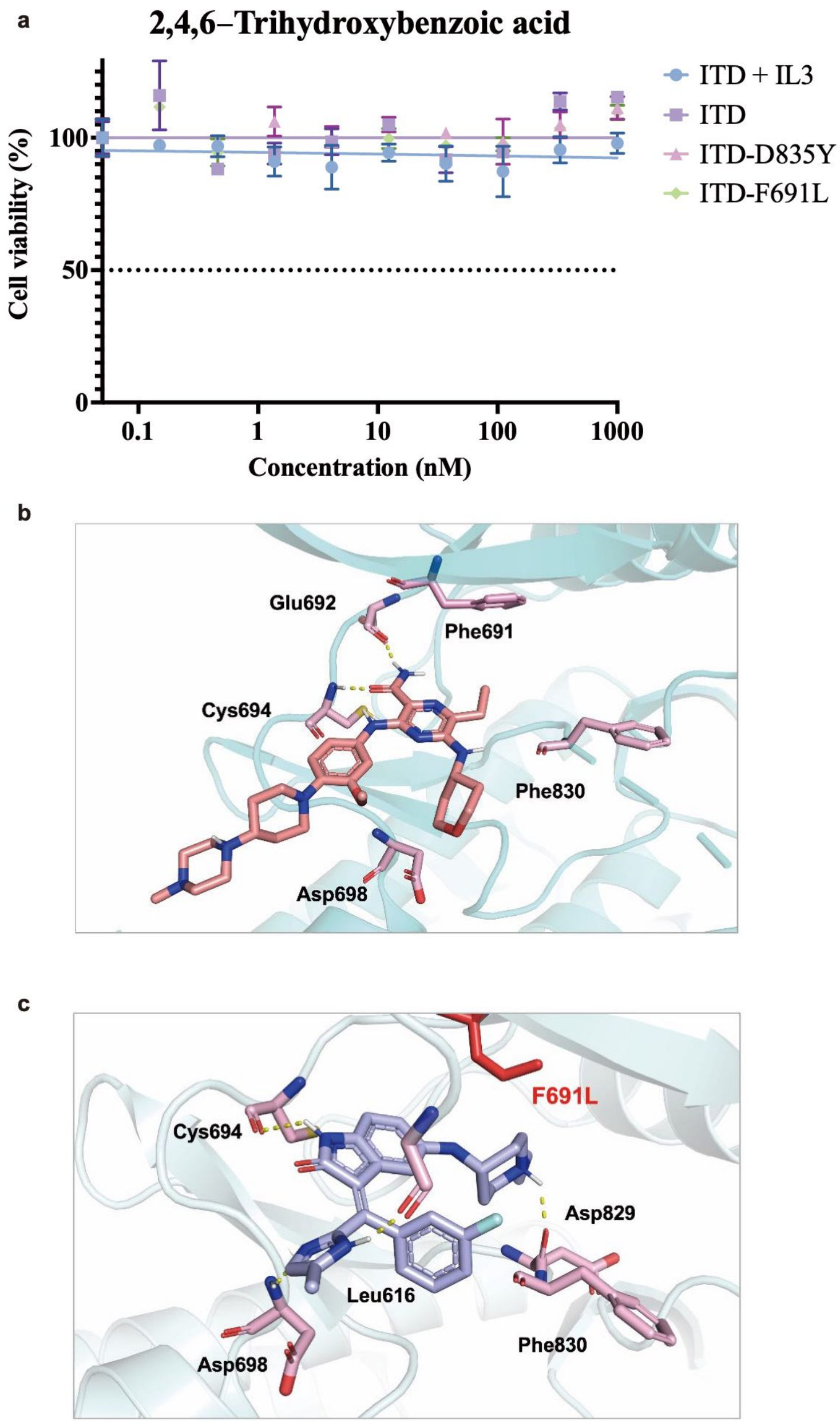

Supplementary Figure 2. Evaluation of a negative control compound and molecular docking analysis of FLT3 inhibitors. (a) Dose–response curves of Ba/F3 cells expressing FLT3-ITD (in the absence or presence of interleukin-3), FLT3-ITD-D835Y, or FLT3-ITD-F691L following 48 h of treatment with 2,4,6-trihydroxybenzoic acid. No cytotoxic or inhibitory activity was observed across all tested cell lines. Data are presented as mean ± SD from three independent experiments. (b) Molecular docking model of gilteritinib within the ATP-binding pocket of FLT3-ITD, generated using the crystal structure of FLT3 (PDB code: 6JQR) as a template. (c) Molecular docking model of XL999 in complex with FLT3-ITD-F691L, based on PDB entry 6JQR, illustrating the binding orientation of XL999 in proximity to the gatekeeper residue F691L and surrounding residues, including Cys694, Asp698, Leu616, and Asp829.

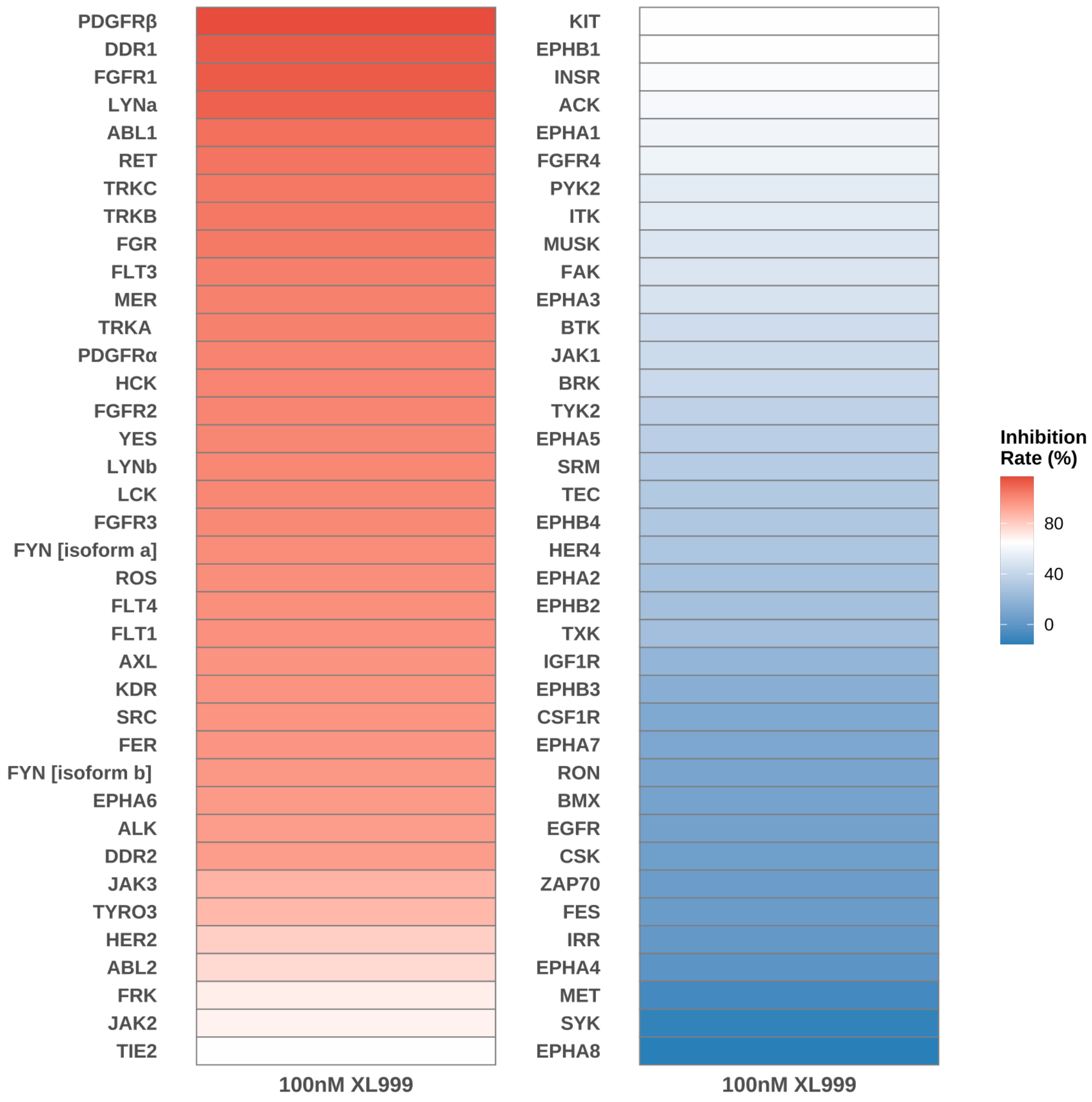

Supplementary Figure 3. Kinase selectivity profiling of XL999. Ranked heatmap illustrating the percentage inhibition across a broad human kinase panel in the presence of 100 nM XL999.

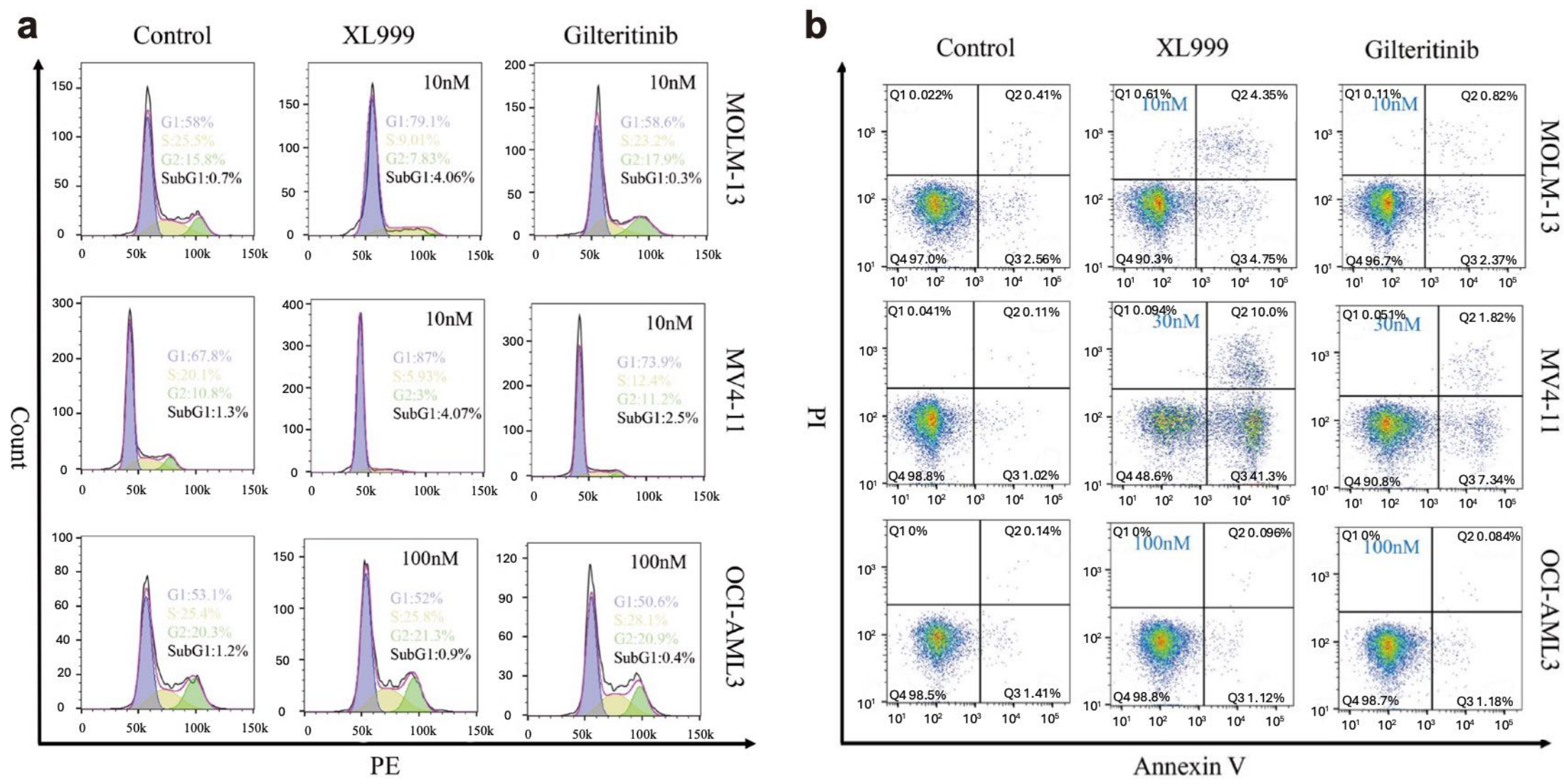

Supplementary Figure 4. Effects of XL999 on cell cycle distribution and apoptosis in AML cells. (a) Representative flow cytometric histograms showing cell cycle distribution in MOLM-13, MV4-11, and OCI-AML3 cells following 24 h of treatment with vehicle control (DMSO), gilteritinib, or XL999 at the indicated concentrations. (b) Representative flow cytometry dot plots (Annexin V/propidium iodide staining) showing apoptosis induction in MOLM-13, MV4-11, and OCI-AML3 cells after 48 h of treatment with DMSO, gilteritinib, or XL999.

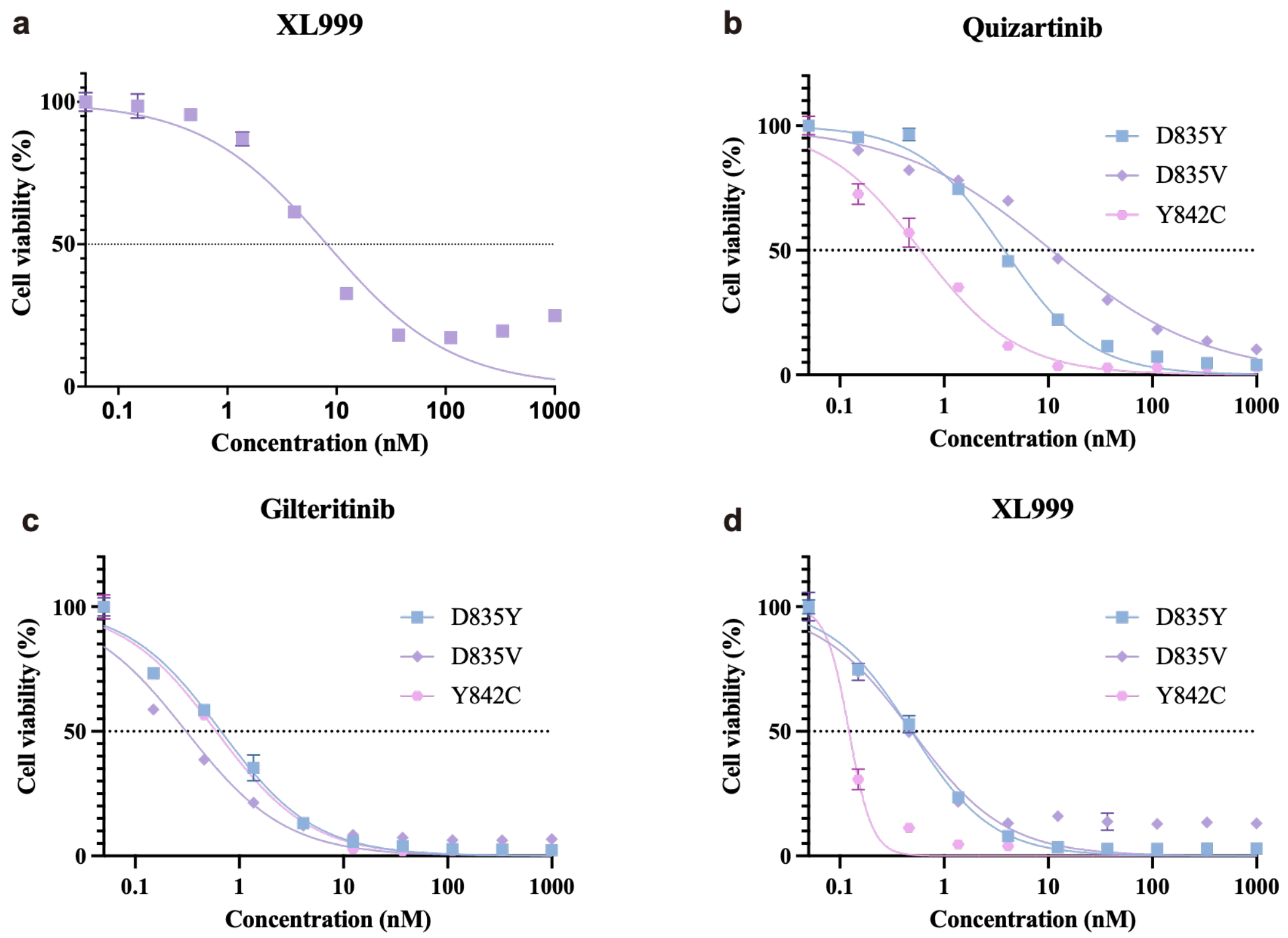

Supplementary Figure 5. Sensitivity profiles of XL999 and reference inhibitors in wild-type and TKD-mutant FLT3-expressing cells. (a) Cell viability dose–response curves of Ba/F3 cells expressing human wild-type FLT3 (FLT3-WT) following 48 h of treatment with XL999 in the presence of 100 nM FLT3 ligand (FL). (b–d) Dose–response curves of Ba/F3 cells expressing individual FLT3 tyrosine kinase domain (TKD) mutations (D835Y, D835V, or Y842C) following 48 h of treatment with escalating concentrations of quizartinib (b), gilteritinib (c), or XL999 (d). Data are presented as mean ± SD.

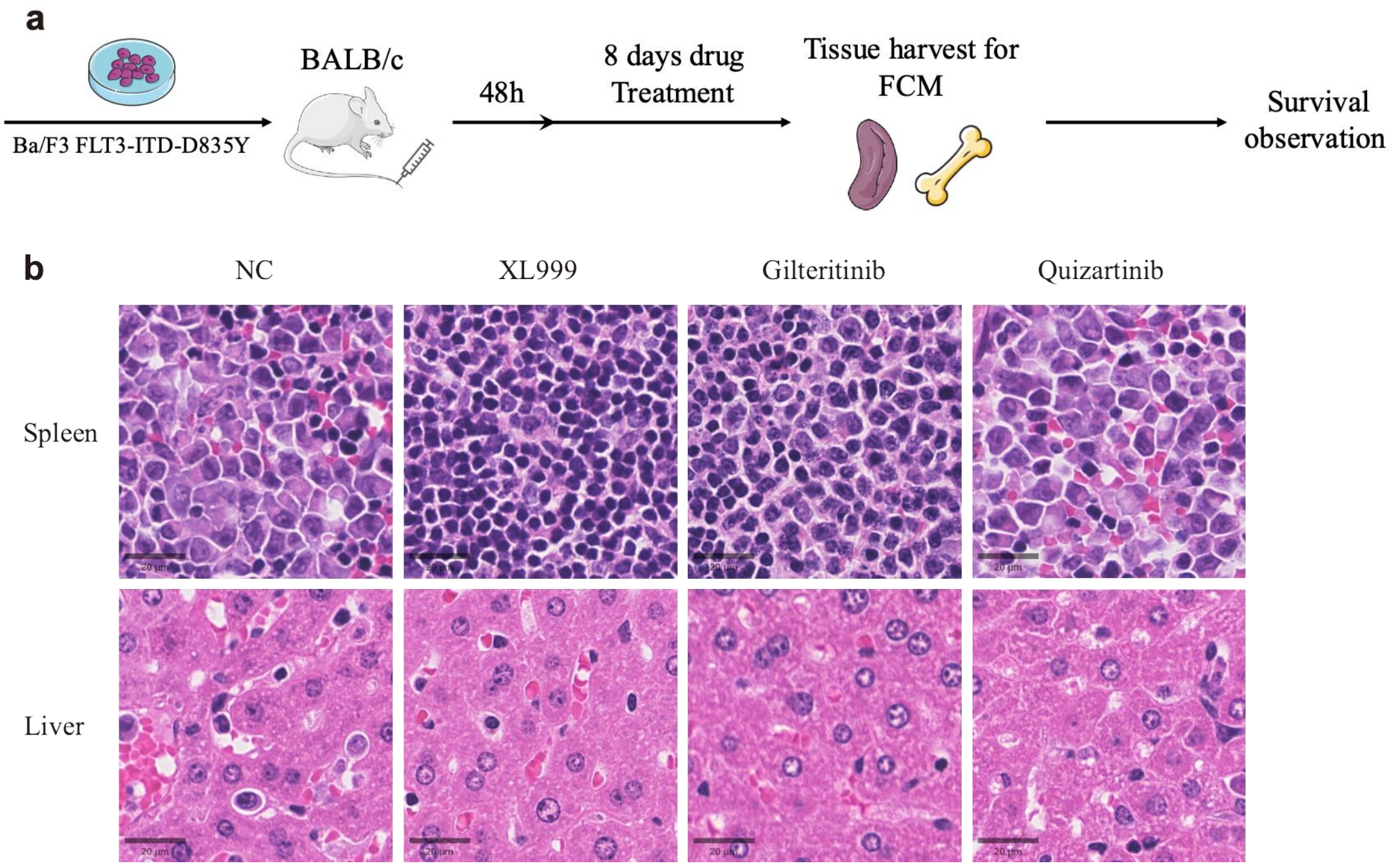

Supplementary Figure 6. Experimental workflow and histopathological evaluation of the Ba/F3-FLT3-ITD-D835Y model. (a) Schematic representation of the experimental workflow, including cell inoculation, drug administration schedule, tissue collection for flow cytometric analysis, and survival monitoring. (b) Representative hematoxylin and eosin (H&E)–stained sections of spleen and liver tissues harvested from Ba/F3-FLT3-ITD-D835Y mice treated with vehicle control (NC), XL999, gilteritinib, or quizartinib (n = 3 mice per group). Scale bars, 20 μm.

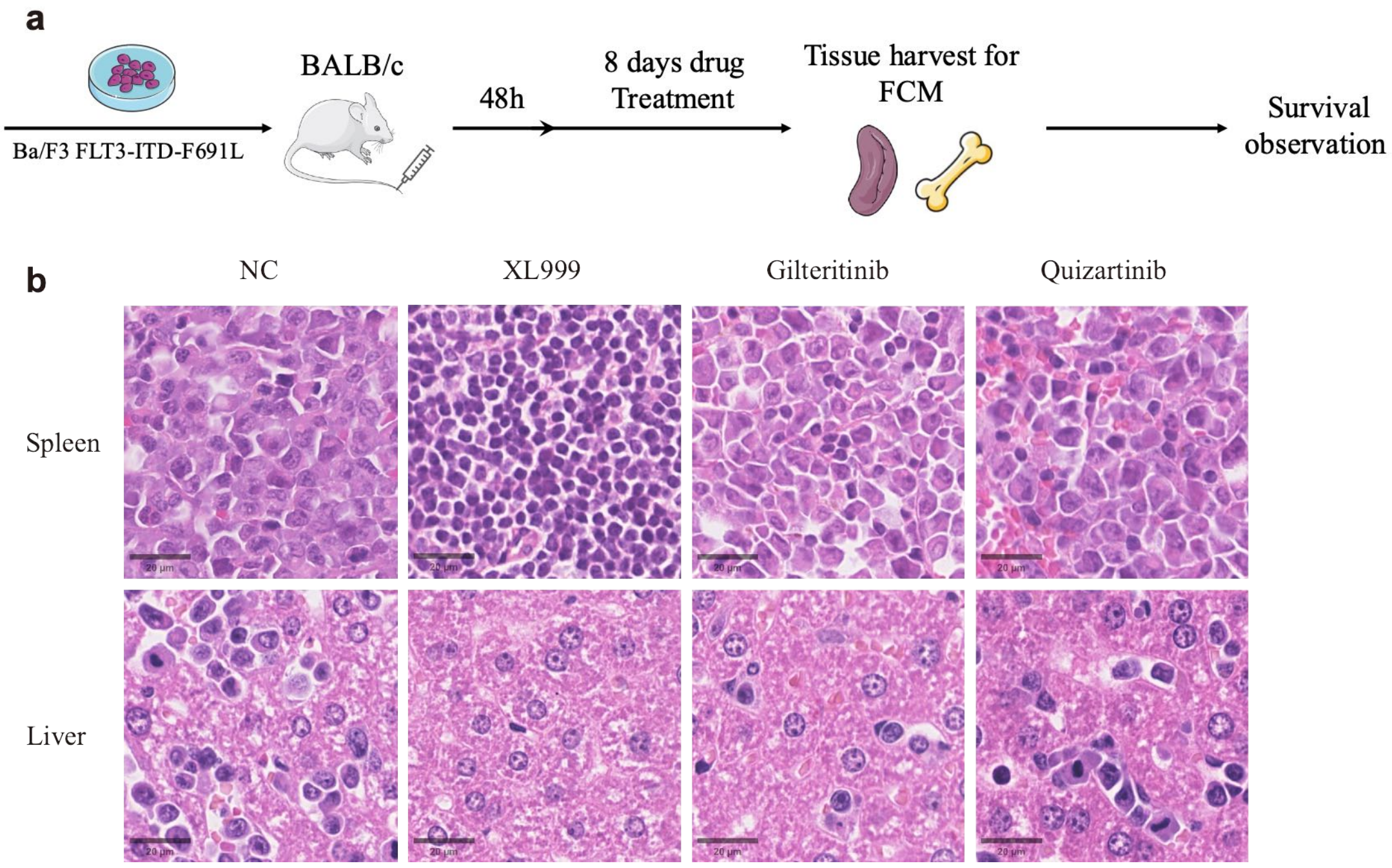

Supplementary Figure 7. Experimental workflow and histopathological evaluation of the Ba/F3-FLT3-ITD-F691L model. (a) Schematic representation of the experimental workflow, including cell inoculation, drug administration schedule, tissue collection for flow cytometric analysis, and survival monitoring. (b) Representative hematoxylin and eosin (H&E)–stained sections of spleen and liver tissues harvested from Ba/F3-FLT3-ITD-F691L mice treated with vehicle control (NC), XL999, gilteritinib, or quizartinib (n = 3 mice per group). Scale bars, 20 μm.

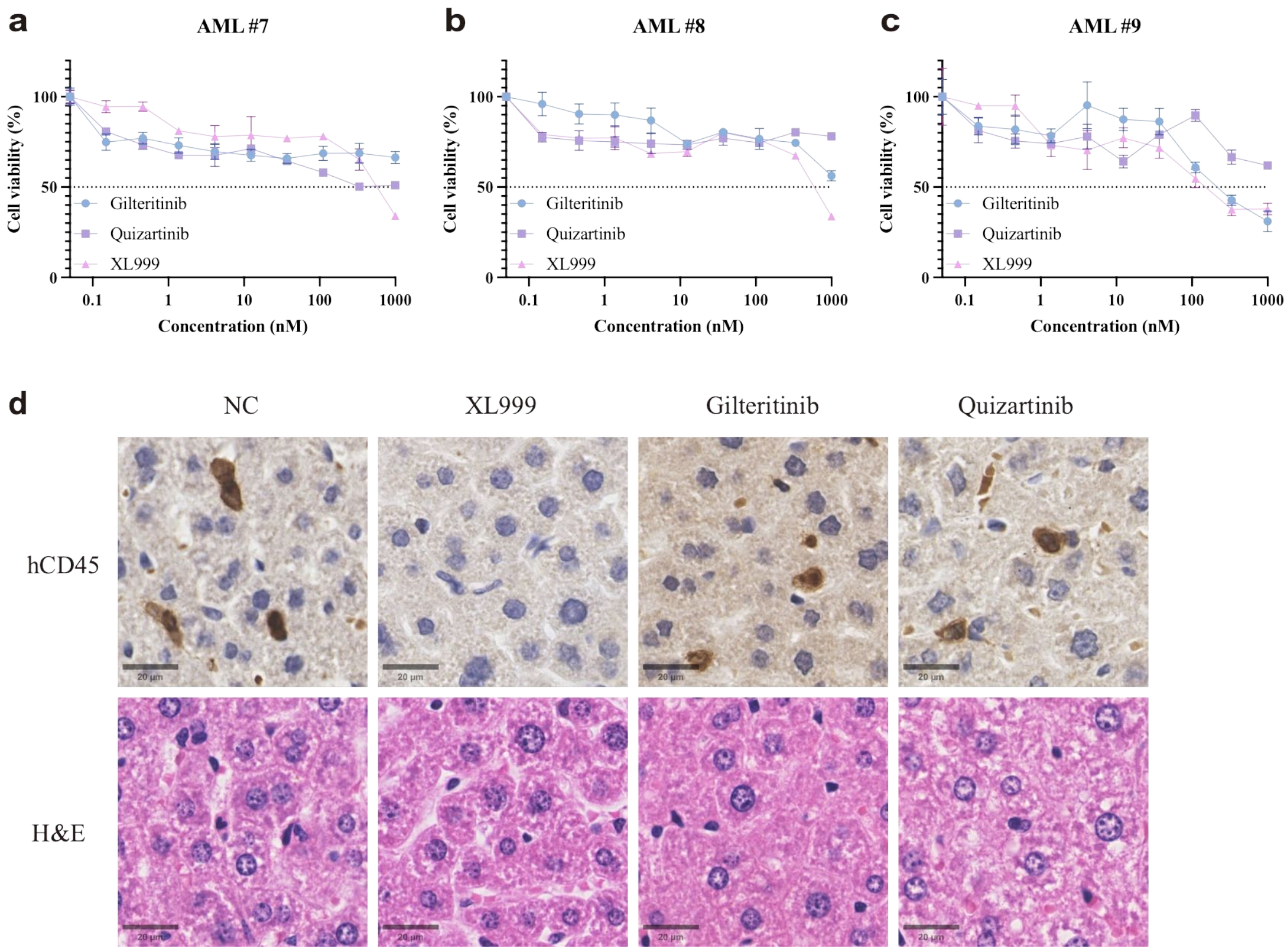

Supplementary Figure 8. Ex vivo antileukemic activity of XL999 on primary AML blasts and histopathological analysis of PDX liver tissues. (a–c) Ex vivo dose–response curves of primary human AML blasts isolated from FLT3-ITD–positive patients following 48 h of treatment with increasing concentrations of XL999, gilteritinib, or quizartinib. Data are presented as mean ± SD. (d) Representative immunohistochemical (IHC) staining of human CD45 (hCD45, upper panels) and corresponding hematoxylin and eosin (H&E) staining (lower panels) in liver sections from PDX mice under the indicated treatment conditions (n = 3 mice per group). Scale bars, 20 µm.

Supplementary Table 1: Measured c-t data of XL999 compound (concentration: ng·ml⁻¹): Intravenous administration (3 mg/kg)

t	No.1	No.2	No.3	Mean	SD
0.083	248.33	232.48	275.05	251.953	21.515
0.25	212.83	223.51	240.56	225.633	13.986
0.5	138.37	140.24	188.78	155.797	28.58
2	123.44	101.71	89.38	104.843	17.245
4	16.85	25.12	31	24.323	7.109
6	9.76	11.87	12.93	11.52	1.614
8	5.23	5.33	9.84	6.8	2.633
24					
48					

Note: t: time after administration (h); No.1–No.3 represent individual experimental rats; Mean: average plasma concentration; SD: standard deviation. Empty cells indicate concentrations below the lower limit of quantification (LLOQ).

Supplementary Table 2: Measured c-t data of XL999 compound (concentration: ng·ml⁻¹): Oral administration (30 mg/kg)

t	No.1	No.2	No.3	Mean	SD
0.083	9.99	14.23	20.98	15.067	5.543
0.25	28.18	44.52	67.12	46.607	19.554
0.5	52.35	50.52	59.72	54.197	4.87
2	53.69	40.73	37.47	43.963	8.58
4	116.66	135.36	157.84	136.62	20.619
6	100.71	139.18	133.81	124.567	20.834
8	72.05	41.47	53.64	55.72	15.396
24	3.04	2.14			
48					

Note: t: time after administration (h); No.1–No.3 represent individual experimental rats; Mean: average plasma concentration; SD: standard deviation. Empty cells indicate concentrations below the lower limit of quantification (LLOQ).

Supplementary Table 3: Results of XL999 compound processed by DAS 2.0 software: Intravenous administration (3 mg/kg)

Parameter	Unit	No.1	No.2	No.3	Mean	SD
AUC(0-t)	ug/L*h	482.934	467.101	516.225	488.753	25.074
AUC(0-∞)	ug/L*h	501.049	480.97	526.237	502.752	22.682
AUMC(0-t)		800.007	817.442	892.694	836.714	49.257
AUMC(0-∞)		1004.193	962.941	1096.022	1021.052	68.123
MRT(0-t)	h	1.657	1.75	1.729	1.712	0.049
MRT(0-∞)	h	2.004	2.002	2.083	2.03	0.046
VRT(0-t)	h^2	2.379	2.815	3.253	2.816	0.437
VRT(0-∞)	h^2	5.991	5.091	5.834	5.639	0.481
t1/2z	h	2.369	1.788	1.424	1.86	0.477
Tmax	h	0.083	0.083	0.083	0.083	0
CLz	L/h/kg	5.987	6.237	5.701	5.975	0.268
Vz	L/kg	20.471	16.093	11.715	16.093	4.378
Cmax	ug/L	248.33	232.48	275.05	251.953	21.515

Note: Data are presented as Mean ± SD (n = 3). Pharmacokinetic parameters were calculated by non-compartmental analysis using DAS 2.0 software. AUC(0-t): Area under the plasma concentration-time curve from time zero to the last measurable concentration. AUC(0-∞): Area under the plasma concentration-time curve from time zero to infinity. AUMC(0-t): Area under the first moment of plasma concentration-time curve from time zero to the last measurable concentration (expressed in $\mu g/L \cdot h^2$). AUMC(0-∞): Area under the first moment of plasma concentration-time curve from time zero to infinity (expressed in $\mu g/L \cdot h^2$). MRT(0-t): Mean residence time from time zero to the last measurable concentration. MRT(0-∞): Mean residence time from time zero to infinity. VRT(0-t): Variance of residence time from time zero to the last measurable concentration. VRT(0-∞): Variance of residence time from time zero to infinity. t1/2z: Terminal elimination half-life. Tmax: Time to reach the peak plasma concentration. CLz: Total body clearance. Vz: Apparent volume of distribution during the terminal elimination phase. Cmax: Peak plasma concentration.

Supplementary Table 4: Results of XL999 compound processed by DAS 2.0 software: oral administration (30 mg/kg)

Parameter	Unit	No.1	No.2	No.3	Mean	SD
AUC(0-t)	ug/L*h	1254.398	1065.974	771.385	1030.586	243.443
AUC(0-∞)	ug/L*h	1269.979	1076.179	996.613	1114.257	140.605
AUMC(0-t)		8125.471	6316.886	3458.549	5966.969	2353.056
AUMC(0-∞)		8577.495	6625.299	5785.708	6996.167	1432.367
MRT(0-t)	h	6.478	5.926	4.484	5.629	1.03
MRT(0-∞)	h	6.754	6.156	5.805	6.238	0.48
VRT(0-t)	h^2	11.358	10.285	3.744	8.462	4.121
VRT(0-∞)	h^2	17.726	15.619	14.829	16.058	1.498
t1/2z	h	3.54	3.447	2.568	3.185	0.536
Tmax	h	4	6	4	4.667	1.155
CLz/F	L/h/kg	23.622	27.876	30.102	27.2	3.292
Vz/F	L/kg	120.669	138.659	111.562	123.63	13.789
Cmax	ug/L	116.66	139.18	157.84	137.893	20.62

Note: Data are presented as Mean ± SD (n = 3). Pharmacokinetic parameters were calculated by non-compartmental analysis using DAS 2.0 software. AUC(0-t): Area under the plasma concentration-time curve from time zero to the last measurable concentration. AUC(0-∞): Area under the plasma concentration-time curve from time zero to infinity. AUMC(0-t): Area under the first moment of plasma concentration-time curve from time zero to the last measurable concentration (expressed in $\mu g/L \cdot h^2$). AUMC(0-∞): Area under the first moment of plasma concentration-time curve from time zero to infinity (expressed in $\mu g/L \cdot h^2$). MRT(0-t): Mean residence time from time zero to the last measurable concentration. MRT(0-∞): Mean residence time from time zero to infinity. VRT(0-t): Variance of residence time from time zero to the last measurable concentration. VRT(0-∞): Variance of residence time from time zero to infinity. t1/2z: Terminal elimination half-life. Tmax: Time to reach the peak plasma concentration. CLz/F: Total body clearance divided by the absolute oral bioavailability (F). Vz/F: Apparent volume of distribution during the terminal elimination phase divided by the absolute oral bioavailability (F). Cmax: Peak plasma concentration.

Absolute Oral Bioavailability (F) Calculation:

The absolute oral bioavailability (F) of compound XL999 in rats was calculated from the mean AUC(0-t) values of oral (p.o.) and intravenous (i.v.) administrations using the following formula:

$$F = [\ AUC(po) \times Dose(iv)\ ] / [\ AUC(iv) \times Dose(po)\ ] \times 100\%$$

$$F = [\ 1030.586 \times 3\ ] / [\ 488.753 \times 30\ ] \times 100\% = 21.09\%$$

Supplementary Table 5. Clinical information relevant to AML patient samples

Patient ID	Age	Sex	Sample type	FLT3 Status	Other mutations
#1	63	male	BM	*FLT3*-ITD	NA
#2	65	female	BM	*FLT3*-ITD	*BCOR*
#3	40	female	BM	*FLT3*-ITD	*ASXL1 NF1*
#4	58	female	BM	*FLT3*-ITD	*NPM1 DNMT3A MYC*
#5	16	male	BM	*FLT3*-ITD	*KMT2A-PTD*
#6	49	male	BM	*FLT3*-ITD	*BCOR CEBPA*
#7	58	male	BM	*FLT3*-ITD	*NPM1 DNMT3A*
#8	50	male	BM	*FLT3*-ITD	*CEBPA TET2*
#9	36	male	BM	*FLT3*-ITD	*DNMT3A*

Note: Clinical characteristics and mutational profiles of primary blasts isolated from nine patients diagnosed with acute myeloid leukemia (AML). These primary samples were utilized for ex vivo drug sensitivity assays and translational validation in this study. Notably, the primary bone marrow sample from Patient #1 was specifically utilized for the establishment of the in vivo patient-derived xenograft mouse model. Abbreviations: AML Acute myeloid leukemia, BM Bone marrow, FLT3-ITD FMS-like tyrosine kinase 3 internal tandem duplication; KMT2A-PTD Lysine methyltransferase 2A partial tandem duplication; NA: Not available.
